# Phytosynthesis of silver nanoparticles using aqueous sandalwood (*Santalum album* L.) leaf extract: Divergent effects of SW-AgNPs on proliferating plant and cancer cells

**DOI:** 10.1371/journal.pone.0300115

**Published:** 2024-04-25

**Authors:** Archana Gowda, Suman T. C., Veena S. Anil, Swetha Raghavan

**Affiliations:** 1 Department of Plant Biotechnology, University of Agricultural Sciences, GKVK, Bangalore, India; 2 National Centre for Biological Sciences, Bangalore, India; REVA University, INDIA

## Abstract

The biogenic approach for the synthesis of metal nanoparticles provides an efficient eco-friendly alternative to chemical synthesis. This study presents a novel route for the biosynthesis of silver nanoparticles using aqueous sandalwood (SW) leaf extract as a source of reducing and capping agents under mild, room temperature synthesis conditions. The bioreduction of Ag^+^ to Ag^o^ nanoparticles (SW-AgNPs) was accompanied by the appearance of brown color, with surface plasmon resonance peak at 340-360 nm. SEM, TEM and AFM imaging confirm SW-AgNP’s spherical shape with size range of 10-32 nm. DLS indicates a hydrodynamic size of 49.53 nm with predominant negative Zeta potential, which can contribute to the stability of the nanoparticles. FTIR analysis indicates involvement of sandalwood leaf derived polyphenols, proteins and lipids in the reduction and capping of SW-AgNPs. XRD determines the face-centered-cubic crystalline structure of SW-AgNPs, which is a key factor affecting biological functions of nanoparticles. This study is novel in using cell culture methodologies to evaluate effects of SW-AgNPs on proliferating cells originating from plants and human cancer. Exposure of groundnut calli cells to SW-AgNPs, resulted in enhanced proliferation leading to over 70% higher calli biomass over control, enhanced defense enzyme activities, and secretion of metabolites implicated in biotic stress resistance (Crotonyl isothiocyanate, Butyrolactone, 2-Hydroxy-gamma-butyrolactone, Maltol) and plant cell proliferation (dl-Threitol). MTT and NRU were performed to determine the cytotoxicity of nanoparticles on human cervical cancer cells. SW-AgNPs specifically inhibited cervical cell lines SiHa (IC_50_–2.65 ppm) and CaSki (IC_50_–9.49 ppm), indicating potential use in cancer treatment. The opposing effect of SW-AgNPs on cell proliferation of plant calli (enhanced cell proliferation) and human cancer cell lines (inhibition) are both beneficial and point to potential safe application of SW-AgNPs in plant cell culture, agriculture and in cancer treatment.

## Introduction

Nanotechnology deals with materials at nanoscale, their synthesis, and their perpetually expanding range of applications [1, 2]. Nanoparticles reveal completely new or improved properties based on specific characteristics such as size, distribution and morphology, when compared with larger particles of the bulk material they are made of. These distinct properties mainly arise due to their higher surface area to volume ratio. Their concomitant unique properties thus have applications in medicine, catalysis [3, 4], drug delivery [5], environmental remediation, renewable energy, water treatment, hazardous chemical substitution, and waste management [6, 7] and various potential applications in agriculture.

Nanoparticles can be synthesized by chemical, physical, and biological synthesis methods. Physical and chemical synthesis of nanoparticles requires high energy inputs, including pressure and temperature, some involve the use of hazardous chemicals or release hazardous waste [8]. The use of biological entities such as bacteria [9], fungus [10], algae [7, 11], enzyme [12], marine oyster [4] and plant extracts in the synthesis of nanoparticles is gaining notable interest as biological methods are safe, economical, amenable to scale-up, and eco-friendly processes. Synthesis of nanoparticles by using plant extracts (phytosynthesis) has added advantages over the microorganisms-mediated synthesis because it eliminates the maintenance of microbial cultures, and also provides unique plant derived secondary metabolites for nanoparticle stabilization [13]. In addition, plants are easily accessible, extensively dispersed and safe to handle [14]. Studies have suggested that biomolecules like protein, phenols, flavonoids and other phytochemicals have the ability to reduce metallic ions to the nano size and also play an important role in the capping of the nanoparticles for stability. Thus, phytosynthesis of biocompatible, and biofunctionalized metal nanoparticles thus deserve credit as a simple, rapid, non-toxic, cost-effective, eco-friendly, and safe method [15–17].

Noble metals such as gold, platinum, silver, titanium, and palladium have been used extensively to synthesize nanomaterials [18–20]. Silver nanoparticles (AgNPs), have gained importance because of their special properties in terms of surface morphology, size distribution, particle composition, particle reactivity in the solution, efficiency of the ion release, electronic, thermal, optical, magnetic, and catalytic features [21–23]. In addition, AgNPs have antimicrobial, anticancer, antiviral, anti-inflammatory and anti-angiogenesis properties [20, 24–27]. These physical, chemical and bioactive properties of AgNPs result in wide applications in food industry, cosmetics, electronics, bio molecular detection, catalysis, biosensors, medicine and agriculture. Indeed, silver is the most commercialized nano material with five hundred ton production per year [28]. Silver nanoparticles are found to be well tolerated in humans, but most effective against bacteria, virus and other micro-organism.

Cancers are cells that have a tendency to proliferate uncontrollably and various forms of such cancers affect a large number of individuals globally. Cervical cancer in particular, is one of the leading causes of death especially in Indian women. The treatment strategies used to tackle these cancers include radiotherapy, chemotherapy with platinum-based drugs apart from surgery and targeted therapy where suitable [29]. Plant based derivatives have been important sources in the discovery and development of critical anticancer agents including taxanes and camptothecin [30]. Nanotechnology can add an important dimension to developing and improving drug-based treatment strategies by enhancing parameters including effectiveness and bioavailability. Nanotechnology can be harnessed for the targeted delivery of therapeutic drugs or dyes for imaging of specific cancer cells [31]. Further, several metal nanoparticles are reported to be specifically inhibitory to cancer cells, thus they have potential in the direct targeted killing of cancerous cells, with minimum side effects when compared to other chemotherapies [17, 32, 33]. Silver nanoparticle along with various plant extracts can have an immense potential to have anticancer activities and explored as a treatment for cancers [34].

Sandalwood is a tree species belonging to the family *Santalaceae* and genus *Santalum*. Sandalwood is indigenous to India and found both in forest ecosystems and in cultivated land. The wood from this tree is highly valued with a characteristic aromatic fragrance. Sandalwood possess a plethora of bioactive compounds such as essential oil and its components (α-santalol and β-santalol), phenolic compounds, terpenoids, fatty acids, β –sitosterols, which contribute towards biological activities and health-promoting effects in humans. Pre-clinical and clinical studies have shown the role of sandalwood extract as antioxidant, anti-inflammatory, antibacterial, antifungal, antiviral, neuroleptic, antihyperglycemic, antihyperlipidemic, and anticancer [35–38]. Alpha-santalol is a component of sandalwood heartwood that is known to have several therapeutic activities including antibacterial and anticancer properties [39–41]. Apart from this, extracts of the sandalwood leaves have also been shown to produce antibacterial activity [42]. Bisabolene a sesquiterpenoid having anticancer activity, is a major compound in the essential oil extracted from *Santalum album* L. The promoter of bisabolene synthetase (*SaBS*) a key enzyme for the synthesis of bisabolene has been shown to drive expression in stem tissue, followed by leaves and flowers [43]. The use of sandalwood leaf extract for the bioreduction and stabilization of Ag nanoparticles would combine the beneficial properties of AgNPs and of sandalwood extracts to result in superior bioactive, bioavailable and biocompatible nanoparticles.

This study deploys a biogenic approach for the synthesis of silver nanoparticles, using aqueous *Santalum album* L. mature leaf extract for the bio reduction of Ag^+^ to Ag nanoparticles (SW-AgNPs). The simple phytosynthesis method developed involves aqueous extraction of leaf metabolites, followed by room temperature bioreduction with minimum stirring. Biogenic approach provides an efficient eco-friendly alternative to chemical synthesis moreover the simple methodology is congenial with economics of future scale-up and commercial synthesis of SW-AgNPs. The study further characterizes SW-AgNPs using SEM, EDAX, UV-Spectroscopy, FTIR, AFM, TEM, DLS and XRD. Further, the aim of this study was to assess the effect of SW-AgNPs on proliferation in two different setting- in plant calli cells and in human cancer cells. *Santalum album* extracts have several therapeutic benefits and in combination with nanotechnology, it can add the dimension of enhanced effectiveness and bioavailability. This study is novel in demonstrating SW-AgNPs-induced cell proliferation and defense-like response in groundnut calli. The study also demonstrates the inhibitory effects of SW-AgNPs on human cervical cell lines. Together the findings implicate SW-AgNP’s potential safe application in tissue culture, agriculture and cancer therapy.

## Results and discussion

### Screening of plant species for high levels of phenolics and flavonoids

Easily available medicinal plant species and tree species were initially screened for their phenol and flavonoid content in order to select a species for the phytosynthesis of AgNPs. Total phenolic and flavonoids were extracted and estimated using methods described in Materials and Methods. Table 1 represents total phenolic and flavonoid content in leaves of Neem (*Azadirachta indica* A. Juss), Amrutha balli (*Tinospora cordifolia* (Willd.) Hook. f. and Thoms.), Sandalwood (*Santalum album* L.), Brahmi (*Bacopa monnieri* L.), Pongamia (*Pongamia pinnata* L.) and tamarind (*Tamarindus indica* L.).

**Table 1:** Screening of plants for levels of phenolics and flavonoids.

Genotypes	Total phenolic content in leaves (mg/g FW)	Total Flavonoid content in leaves (mg/g FW)
Amrutha balli	1.76	7.25
Sandalwood	3.03	19.29
Neem	3.35	20.46
Tamarind	1.56	15.47
Pongemia	0.42	13.78
Brahmi	1.87	15.37
CD@ 1%	1.77	2.34
SEM	0.04	0.75

Unit- mg phenolic per gram fresh weight

**Fig 1:**
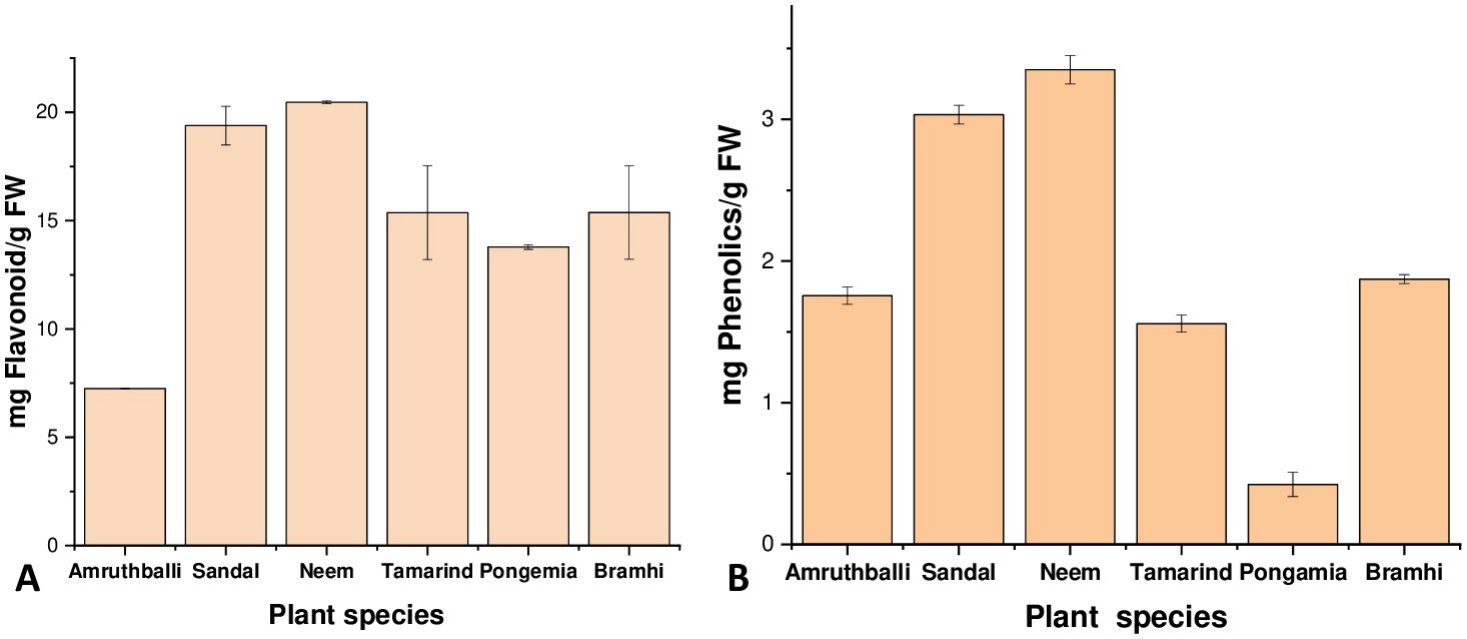
Total flavonoids and phenolics in plant leaves: A, Total flavonoid and B, total Phenolic levels in leaves of Neem (*Azadirachta indica*), Amrutha balli (*Tinospora cordifolia*), Sandalwood (*Santalum album* L.), Brahmi (*Bacopa monnieri* L.), Pongamia (*Pongamia pinnata* L.) and tamarind (*Tamarindus indica* L.).

Among the six species screened, marked higher phenolic and flavonoid content were detected in the leaves of sandalwood and Neem (Fig 1) (Table 1). Phenolics and flavonoids are secondary metabolites in plants that can act as reductants and can contribute to reducing Ag^+^ to sliver nanoparticles. Sandalwood is a medium sized tree with easily accessible flush of leaves that are available throughout the year. Sandalwood is widely distributed tree species in southern India and abundantly present within the University campus. The Sandalwood tree is known for its medicinal properties, having a plethora of biomolecules [38]. Utilizing the leaf extract for the phytosynthesis would result in higher biocompatible and bioactive nanoparticles. Therefore, this study chose to use the aqueous leaf extract of sandalwood for the phytosynthesis of AgNPs.

### Biogenic synthesis of Ag nanoparticles using *Santalum album* L. leaf extract

**Fig 2:**
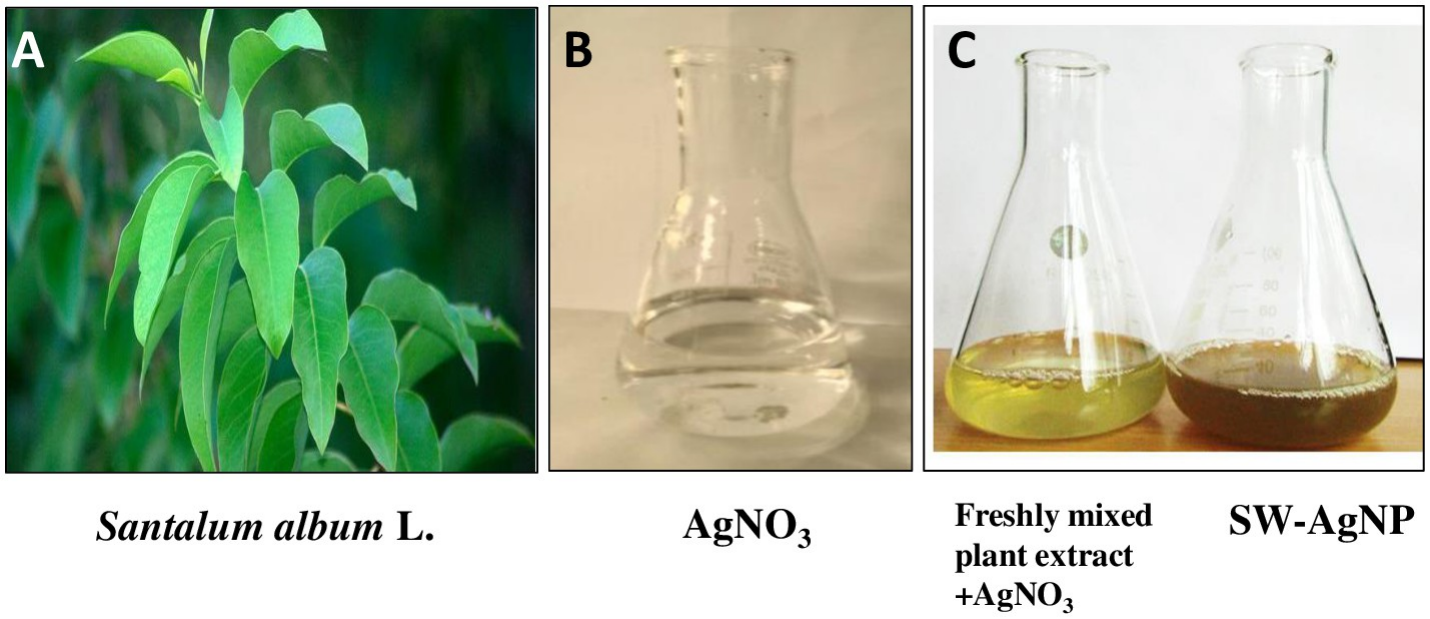
Phytosynthesis of SW-AgNPs using sandalwood aqueous leaf extract: A, Sandalwood leaves; B, AgNO_3_ solution; C, flask on the left- freshly mixed Plant extract and AgNO_3_, and flask on the right- mixture after 8 h-brown colour indicating synthesis of SW-AgNPs.

**Fig 3:**
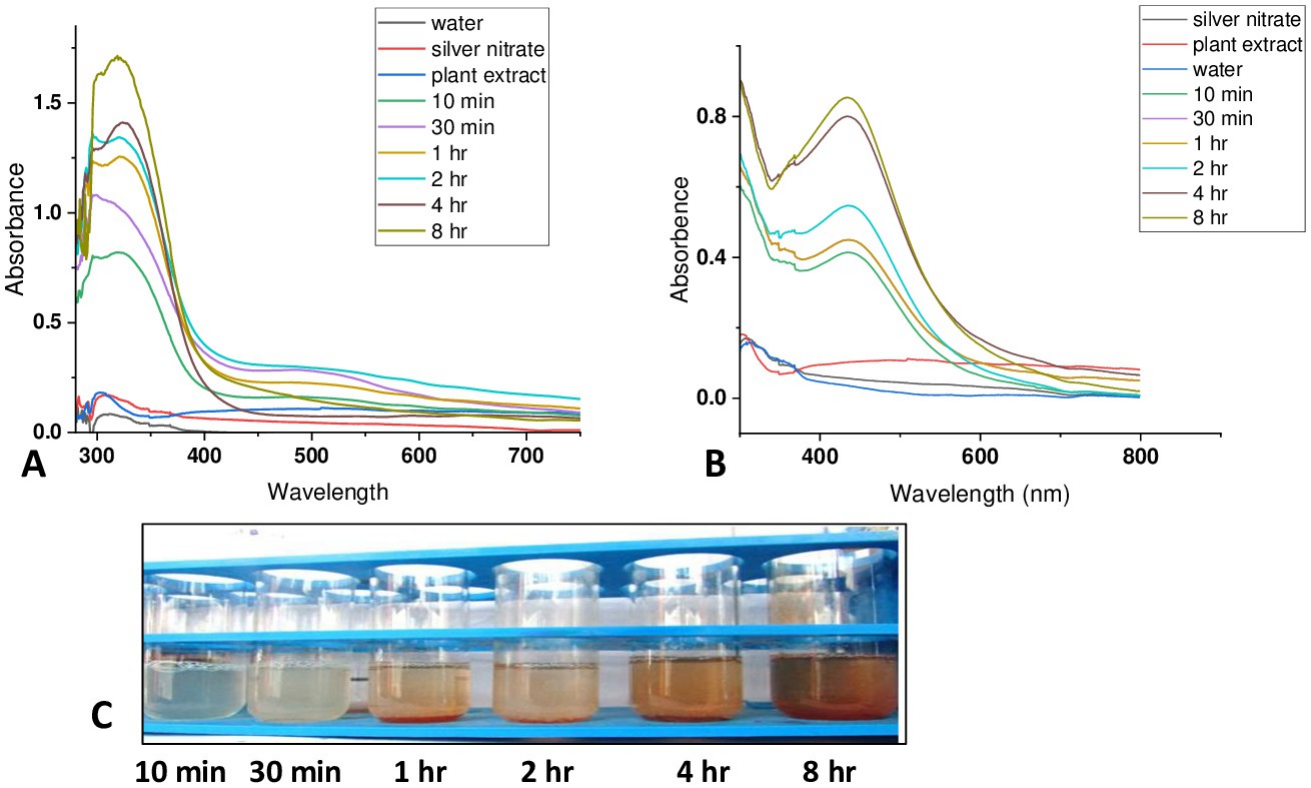
Surface plasmon resonance of SW-AgNPs: The UV spectrum was generated for the mixture of AgNO_3_ and plant extract at increasing reaction times during phytosynthesis. The increase in surface plasmon absorption was recorded as a function of reaction time. Controls included water, AgNO_3_ and plant extract, all three gave negligible absorption peaks compared to the increasing AgNP surface plasmon peak with time. A, phytosynthesis at room temperature; B, Phytosynthesis at 95°C; C, the gradual change in colour of the phytosynthesis mixture during room temperature synthesis.

The healthy mature leaves of sandalwood tree, collected from the University of Agricultural Sciences, Bangalore campus were gently washed with distilled water to remove dust and any other contaminants, and air dried on absorbent blotting sheets. The cleaned leaves were then homogenised and suspended in water, to obtain the aqueous extract of sandalwood leaves. In a typical phytosynthesis procedure, 2 ml of the aqueous plant extract was added to 8 ml of a 1 mM aqueous AgNO_3_ solution. Upon mixing the leaf extract with aqueous solution of the Ag^+^, phytosynthesis was apparent by a colour change of the reaction mixture from a colourless to brown color which intensified with reaction time (Figs 2 and 3C). This colour change arises due to the reduction of Ag^+^ to Ag^o^ and indicates the formation of Ag nanoparticles having surface plasmon absorption. Thus, components of sandalwood leaf extract acted as a reducing agent to convert silver ions (Ag^+^) into nanosilver (Ag^0^), which we term, henceforth, as sandalwood leaf-synthesized silver nanoparticles (SW-AgNPs).

Phytochemicals such as flavonoids, triterpenoids, glycosides, and polyphenolic components might carry out the bioreduction of Ag^+^ to Ag^0^. The mixture was incubated at room temperature or at 95°C for upto 8 h for maximum reduction. The reaction mixture was then centrifuged to pellet down the synthesized SW-AgNPs. The pellet was washed with double distilled water twice, and dried. Alternatively, SW-AgNPs were stored as an aqueous suspension as a concentrated stock.

Incubation (8 hours) of 2:8 (sandalwood leaf extract: 1 mM AgNO_3_ solution) proportionate solution at room temperature results in 400 ppm of AgNPs i.e., from 10 ml of reaction mixture, 4 mg of AgNPs was obtained reproducibly.

### Characterization of silver nanoparticles

#### Characterization of nanoparticles by UV-Vis spectroscopy

As the plant extract was mixed in the aqueous solution of silver nitrate, the color starts changing from colorless/pale green to brown (Fig 2). The brown colour is due to the enhanced optical absorption of synthesized SW-AgNPs. More specifically this intense colour arises from the surface plasmons, which are dipole oscillation arising when an electromagnetic field is coupled to the collective oscillations of conduction electrons [44]. The intensity of brown colour gradually increased as the reaction proceeded and suggested increasing concentration of silver nanoparticles.

UV-Vis spectroscopy is one of the useful methods for characterizing the optical response of metal nanoparticles. This method is quite sensitive to the formation of colloidal metal nanoparticles, due to their intense surface plasmon resonances (SPRs) [45]. Typically, the characteristic formation of colloidal silver nanoparticles is confirmed by the appearance of the sharp SPRs in the range of 350–600 nm [46, 47]. It should be noted that the position of SPRs depends on different factors (e.g., size, shape, etc.). In this study, phytosynthesis of AgNP was confirmed using the increased optical absorption by UV–Vis spectroscopy in the wavelength range of 200–800 nm. The resultant SW-AgNP UV– Vis spectrum (Fig 3A and 3B) showed a peak at 340–360 nm when synthesis was carried out at room temperature and 430–440 nm when synthesis was carried out at 95°C. The absorption peak arises due to surface plasmon vibrations excited by the silver metal lattice. The spectral plasmon peak detected below 450 nm indicates that SW-AgNPs have spherical shape and likely to be less than 50 nm in size. The gradual change in the color of the reaction mixture (Fig 3C) observed with both synthesis processes, was tracked by recording UV-Vis absorption spectra of the aqueous colloidal phytosynthesis solution as the reaction proceeded. A time dependent increase in the absorption peak was obtained both when the synthesis was carried out at room temperature and at 95°C (Fig 3A and 3B). The gradual change in colour intensity of the mixture at room temperature phytosynthesis is shown in Fig 3C. The UV spectral analysis shows that nanoparticles are synthesized within a few hours at both temperatures, however, to ensure maximum reduction of Ag^+^ to Ag the incubation was extended to 8 hours in this study.

#### Fourier transform infrared (FTIR) analysis of SW-AgNPs

The surface of silver nanoparticles generated absorb certain molecules that act as capping agents that stabilize the particles, and protects particles from agglomeration [48]. Phytochemicals such as phenols, terpenoids, flavonoids and proteins can function in capping/stabilizing and reduction of Ag^+^ to Ag nanoparticles. Infra red (IR) spectra is popularly used to determine the naturally present functional groups and molecules in the plants and identify those that show association during nanoparticles phytosynthesis.

**Fig 4:**
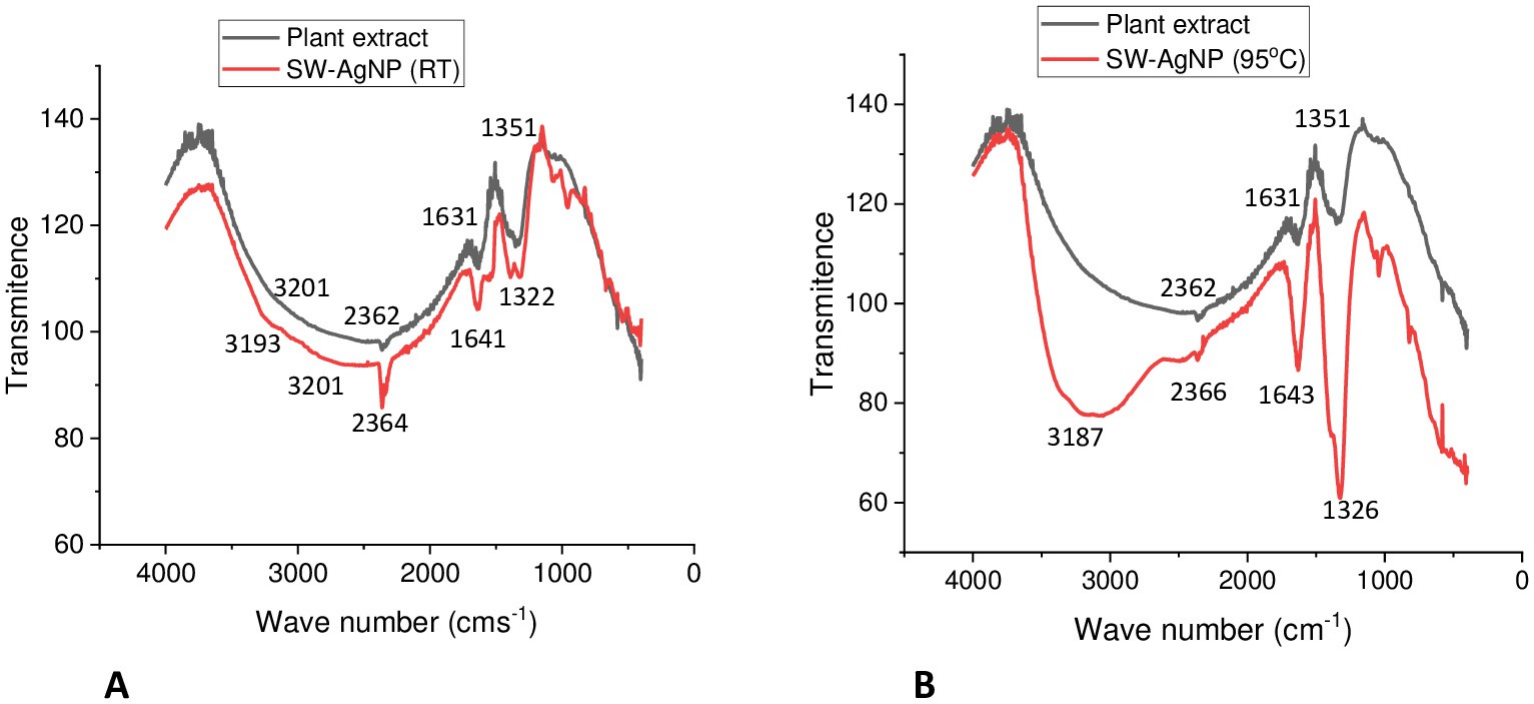
FTIR spectrum of plant extract and SW-AgNPs: A, Phytosynthesis at room temperature and B, Phytosynthesis at 95°C.

In the current study, FTIR analysis (Fig 4) of Sandalwood leaf extract showed peaks at 1351, 1631, 2362, 2784, 3201 cm^1^. The FTIR spectrum of SW-AgNP synthesized at room temperature revealed major peaks at 1322, 1641, 2364, 2800, 3193 cm^1^ (Fig 4A). The SW-AgNP synthesized at 95°C revealed major peaks at 1326, 1643, 2366, 3187, 3070 cm^1^ (Fig 4B). The peaks at 1351, 1322, 1326 cm^1^ represent C-O (carboxylic acid) stretching vibrations of aromatic compounds. The peaks at 1631, 1641, 1643 cm^1^ signify the secondary amide (-C-N) group of peptide bonds, it also indicates stretching of C = O corresponding to aromatic amino groups [49]. The peaks at 2362, 2364, and 2366 cm^1^ signified triple bonded C compounds [50], which are often found in fatty acids of plant origin, and the appearance of these peaks indicates that fatty acids may likely be associated in capping and stabilization of SW-AgNPs. The shift in the peak from 1351 (plant extract) to 1322 cm^1^ (Room temp sysnthesis) and 1326 cm^1^ (95°C synthesis) reveals the participation of aromatic compounds in the synthesis of SW-AgNPs. Shift in the peak from 1631 (Plant extract) to 1641 cm^1^ (Room temp synthesis) and 1643 cm^1^ (95°C synthesis) reveals the involvement of proteins in bioreduction, and capping around the AgNPs. Capped AgNPs are non-agglomerated and stable for long durations. The peaks at 2784 and 2800 cm^1^ are due to the methylene (-C-H) group of aromatic/aliphatic chains [51]. The peaks at 3201, 3187, 3070 cm^1^ correspond to the O-H (hydroxyl) group of flavonoids, triterpenoids, and phenolic compounds. This is an important shift that demonstrates the involvement of polyphenolic compounds in the reduction of Ag^+^ions into SW-AgNPs [52]. The shifting in the bands clearly demonstrate the involvement of polyphenols and proteins in the synthesis (reduction) and stabilization of SW-AgNPs. Phenolic acids possess a phenolic nucleus and a carboxyl group, and thus form a resonance stable phenoxy radical nucleus. Oxidation of phenolic acids leads to stable phenoxy radical formation [53]. Hence, oxidation of phenolic acids by AgNO_3_ produces stable phenoxy radicals and reduced metallic silver atoms. The reduced metallic silver atoms combine together and form a stable capped silver nanoparticle. Sandalwood is reported to have a plethora of phytochemicals including phenolics, fatty acids, β –sitosterol, terpenoids, saponins and flavanols, implicated in medicinal properties [38] and that potentially act as reductant and capping agents during the phytosynthesis demonstrated in this study. The FTIR results were consistent with previous reports, which demonstrate that polyphenolic compounds and proteins are involved in the bioreduction process and also provide stability via capping [49, 51, 52]. This study also indicates the involvement of triple bonded fatty acid in the capping of SW-AgNPs.

Phytosynthesized AgNPs do not require surface coating chemicals for stabilization. In chemically synthesized-AgNPs, these surface coating chemicals lead to the formation of toxic and non-biocompatible AgNPs that cannot be used for various biomedical purposes as they contain toxic/hazardous chemicals on their surface. In contrast, phytosynthesis produces clean, non-toxic, and biocompatible AgNPs that can be used for various biomedical and agricultural purposes. In addition, plant phytochemicals coating the nanoparticle determine the charged state of the nanoparticles, anitioxidant and catalytic properties, and additional novel properties can be imparted by unique plant secondary metabolites. In this study, the bioactive properties of sandalwood extract should add to the bio-effectiveness of resulting nanoparticles.

#### XRD analysis

**Fig 5:**
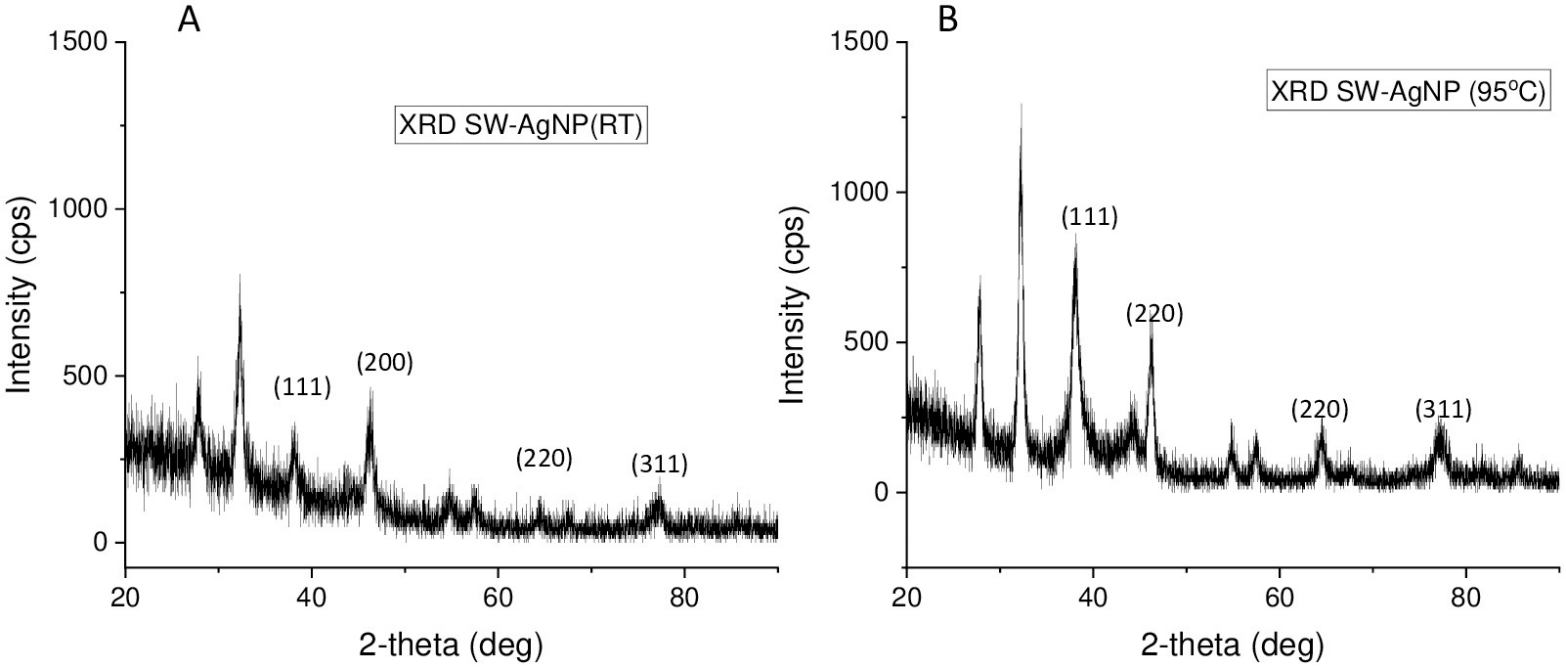
XRD spectra of SWAgNPs: A, Phytosynthesis at room temperature and B, Phytosynthesis at 95°C.

X-ray diffraction (XRD) is valuable characterization tool to prove the formation of AgNPs and determine its crystal structure. The crystal nature of metallic nanoparticles is a key factor affecting their proper biological functions. The XRD pattern of SW-AgNPs phytosynthesized at room temperature showed major distinct diffraction peaks at 2θ of 38.21, 44.18, 64.56, and 77.34 that were indexed to (111), (200), (220), and (311) diffraction planes, respectively (Fig 5A). The XRD pattern of SW-AgNPs phytosynthesized at 95°C also showed major distinct diffraction peaks at 2θ of 38.2, 44.01, 64.56, and 77.04 that were indexed to (111), (200), (220), and (311) diffraction planes, respectively (Fig 5B). The diffraction peaks and their respective planes reveals that the phytosynthesized SW-AgNPs were crystalline with a face-centered cubic (FCC) lattice. The XRD results are consistent with those of previous studies. AgNPs synthesized using *Tithonia diversifolia* [51], Artemisia turcomanica [53] and *Ziziphus Jujuba* [54] are all nanocrystals with FCC structure. A few unassigned peaks observed (Fig 5A and 5B) could be due to the presence of bioorganic compounds/protein(s) from the leaf extract that crystallizes on the surface of the silver or due to AgNO_3_, which might not have been reduced and hence remain in the sample in trace quantity. Such additional XRD peaks have been observed in other reports of phytosynthesized silver nanoparticles [52, 55, 56]. The observed noise in the XRD could be due to very tiny nanoparticles in the synthesized samples, and also from macromolecules derived from plant extract that are responsible for reduction and capping of silver nanoparticles as suggested by Amargeetha and Velavan, 2018 [57].

#### Scanning Electron microscopy (SEM)

**Fig 6:**
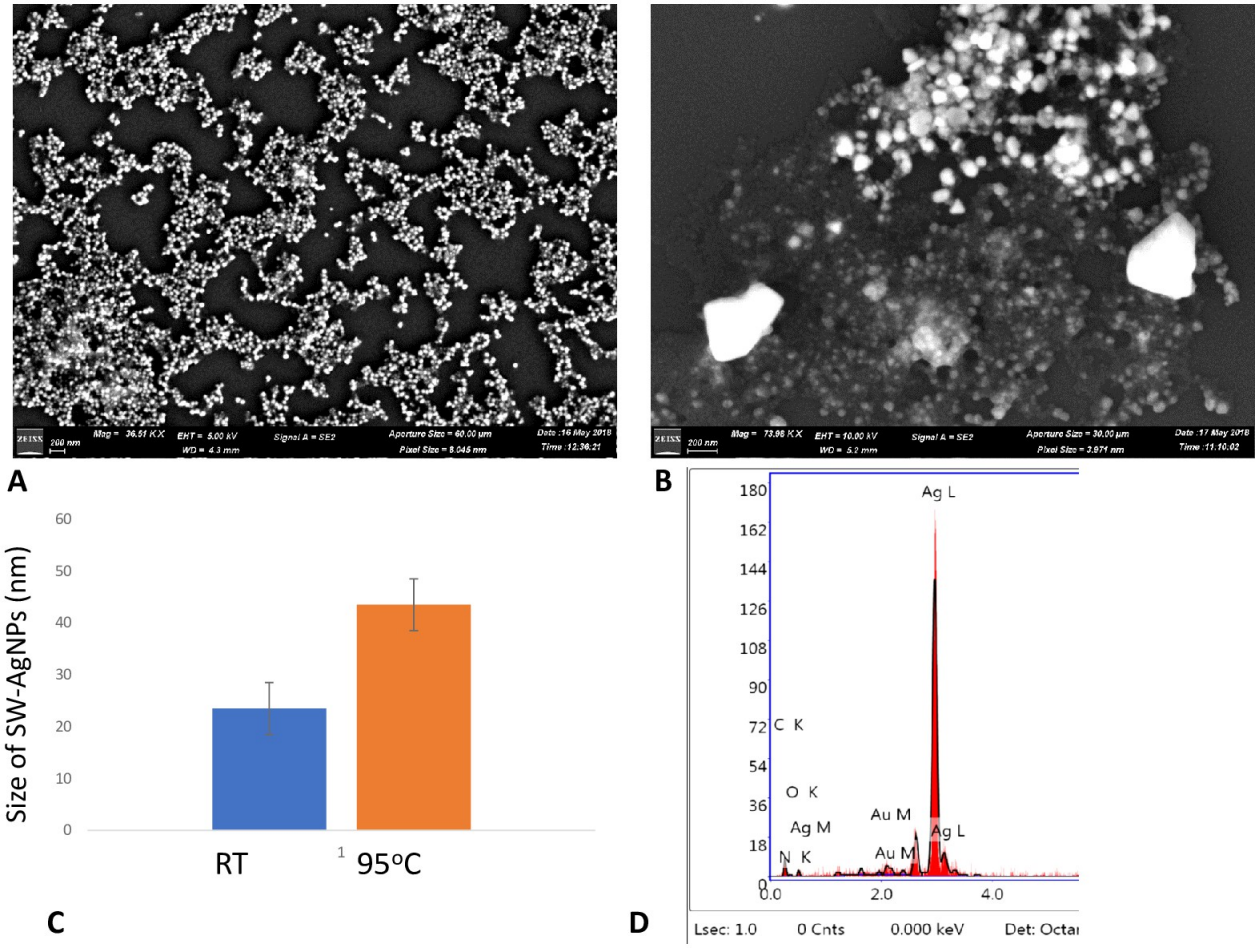
Scanning electron microscope micrograph of SW-AgNPs: A, image of SW-AgNP synthesized at room temperature; B, image of SW-AgNPs synthesized at 95°C; C, size of SW-AgNPs as estimated from SEM images; D, EDAX graph showing typical 3 keV peak for Ag.

The morphological features and size of SW-AgNPs as studied by SEM analysis, indicate that the particles are spherical in shape (Fig 6A and 6B) both when phytosynthesized at room temperature and at 95°C.

By using SEM image data, the size of the nanoparticle was estimated using ImageJ software. SEM images from two different temperature variations, were analyzed by measuring the diameter of 25 AgNPs and the mean with standard error evaluated. The room temperature phytosynthesis produced nanoparticles of 20–25 nm, while the high temperature synthesis produced slightly larger nanoparticle of size range 40–45 nm (Fig 6C). The estimated size may be larger than the actual nanoparticles due to the capping by the proteins and secondary metabolites which is also included during the measurement. It is possible that the convection currents in the reaction mixture caused during high temperature (95°C) reaction conditions, result in continuous mixing of the reaction components, leading to larger sized nanoparticles as compared to synthesis at room temperature, which is kept stationary without stirring for several hours.

SEM imaging was followed by EDX analysis which showed a strong signal at the energy of 3 keV for silver (Fig 6D). The major emission energy at 3 keV, clearly indicates the metal state of the nanoparticles as pure silver.

The SW-AgNPs, synthesized both at 95°C and at room temperature were spherical, and quite comparable with regard to crystalline nature and the compounds making up the capping material, they differed only in average size. As the beneficial effects of nanoparticles are better with smaller nanoparticles, the room temperature nanoparticles can be considered superior compared those obtained at higher temperature. In addition, this study aims to simplify the synthesis process so that up-scaling in future becomes cost effective, and therefore the room temperature synthesized SWAgNP is taken forward for further characterization and bioactivity evaluations.

#### Atomic Force Microscopy (AFM) of SW-AgNPs

**Fig 7:**
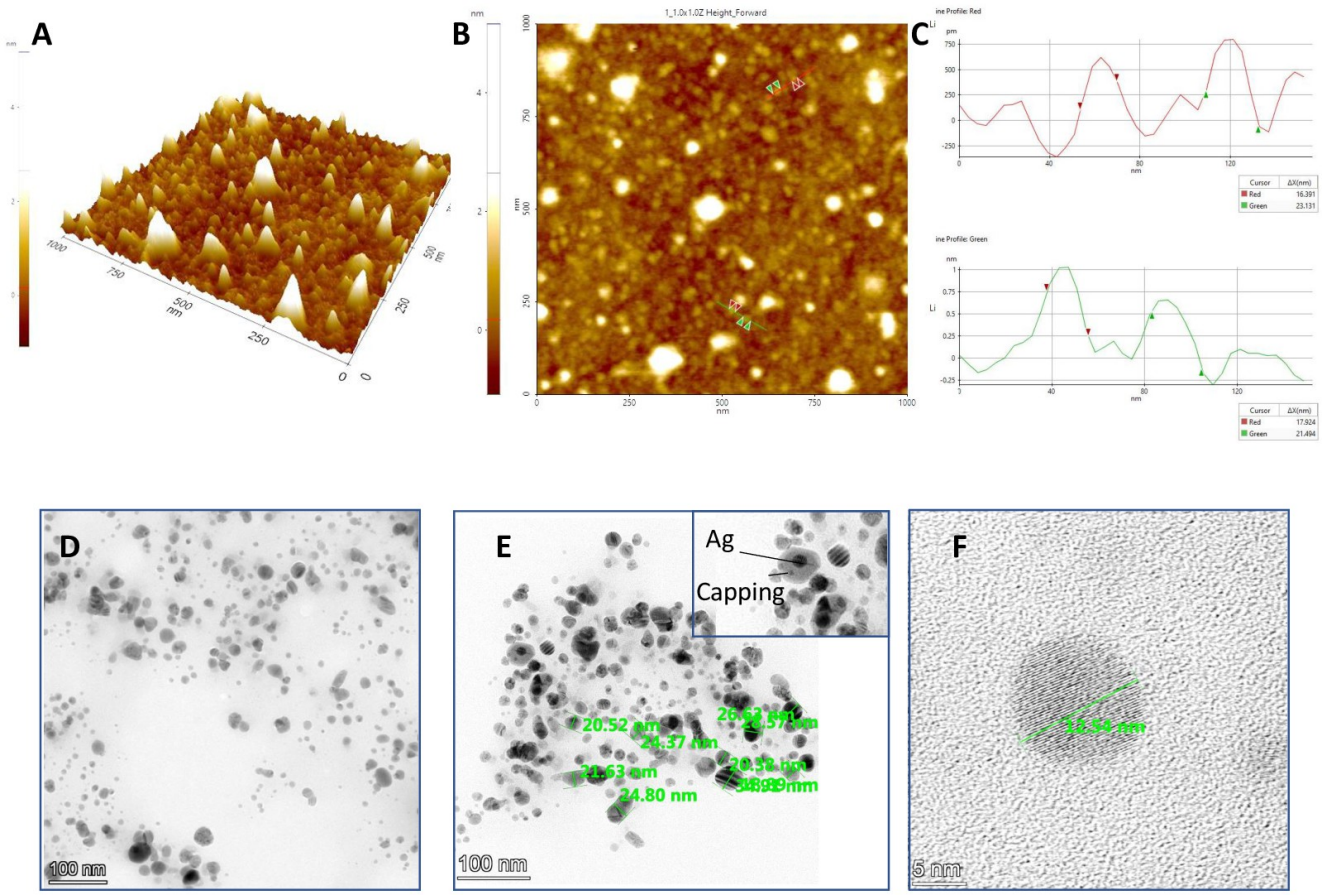
Size, topological features of SW-AgNPs synthesized at Room temperature using AFM and TEM: A, 3-D image of SW-AgNPs obtained from AFM; B, 2-D image of SW-AgNPs; C, graph showing size of nanoparticles as assessed from the 2D AFM image; D, TEM image showing spherical nanoparticles E, TEM image showing size measurements of SWAgNPs, inset shows silver nanoparticles surrounded by capping material; F, high magnification TEM image of a single spherical nanoparticle indicating size.

The topological features of synthesized SW-AgNPs as visualized by AFM are shown in Fig 7A–7C. The images clearly show the presence of spherical shaped silver nanoparticles with an average particle size of 17.9 nm to 21.13 nm. The SW-AgNPs contained roughness and root mean square roughness of 0.29 nm and 0.24 nm, respectively. Our results are in conformity with the earlier reports of AgNPs synthesized using various plant species [58–60].

Further TEM (Fig 7D–7F) clearly shows that SW-AgNPs are spherical in shape, with some agglomerates of small grains and mostly dispersed nanoparticles (Fig 7D) of size range 10 to 32 nm (Fig 7E and 7F). The images also show the silver nanoparticle surrounded by less denser material which represents the capping by the plant derived metabolites (Fig 7E inset).

#### Dynamic light scattering and zeta potential

The hydrodynamic particle size distribution and surface charge (ζ-potential) of the biosynthesized SW-AgNPs were measured by the dynamic light scattering (DLS) technique using Zetasizer Nano from Malvern Instruments. The nanoparticle samples were appropriately diluted to reduce the background scattering.

**Fig 8:**
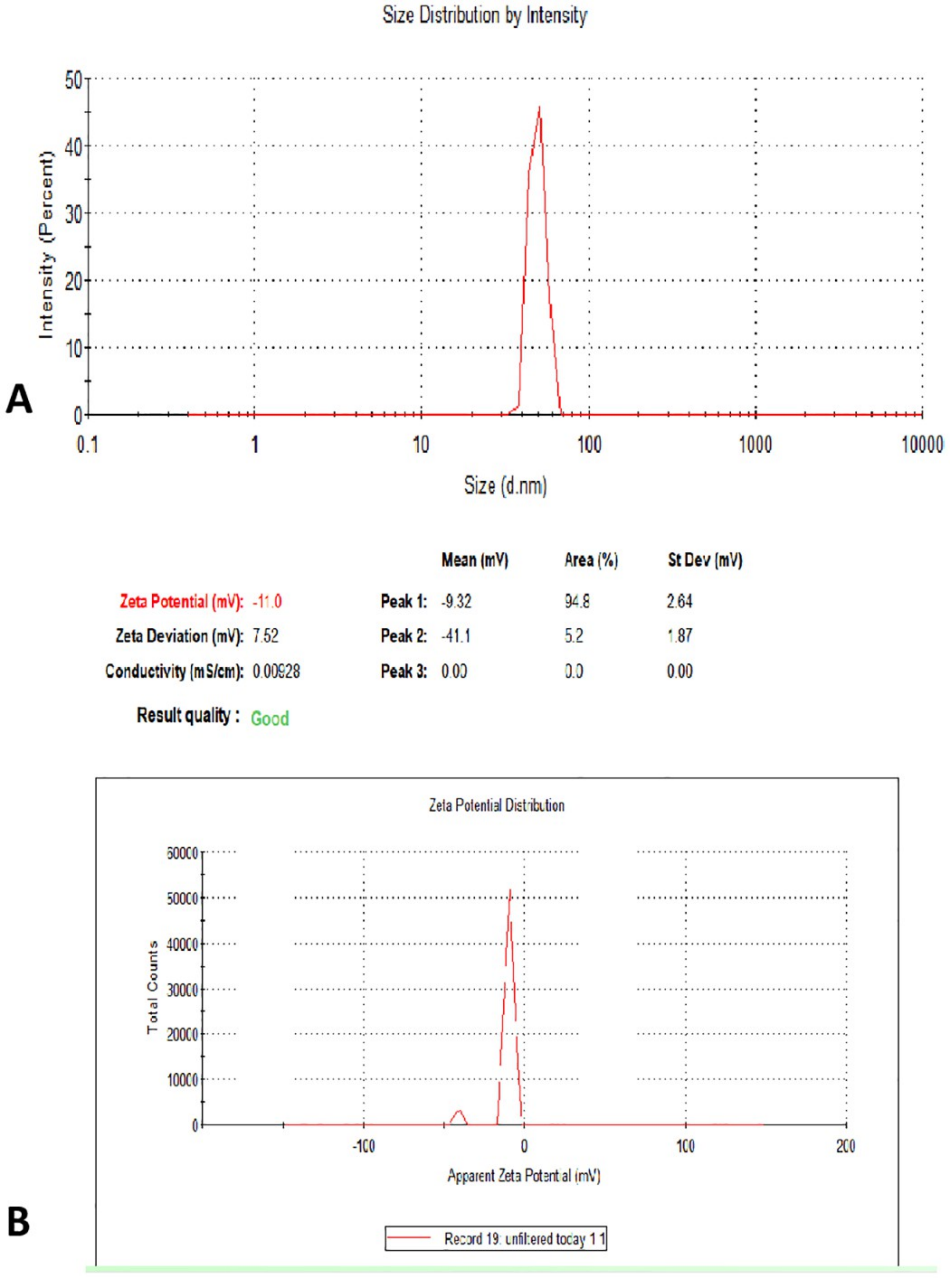
Dynamic light scattering and zeta potential of SW-AgNPs: A, shows the hydrodynamic size distribution of SW-AgNPs by DLS analysis; B, Negative ζ-potential of SW-AgNPs.

The representative DLS size distribution image of SW-AgNPs is shown in Fig 8. In colloidal state, the size distribution of AgNPs ranged from 25 to 70 nm with the calculated average particle size of 49.53 nm (Fig 8A). DLS analyzer shows that the size obtained in colloidal state is higher than size recorded using TEM, AFM and SEM suggesting some aggregation in colloidal state. It is possible that the SW-AgNPs are polydispersed in suspension as the PDI observed is close to 1. However, the hydrodynamic size includes the hydration layer on the surface of AgNPs; thus, this size is generally larger than the size measured from microscopic images. The zeta potential of the SW- AgNPs was found as a major peak at—9.32 mV and another peak at -41.1 mV (Fig 8B). Thus it is inferred that the surface of the nanoparticles is negatively charged and dispersed in the medium. The presence of predominant negative zeta potential implies that there are reactive species in the milieu of the forces of repulsion and attraction among the particles. The higher the negative or positive ζ-potential, the higher the stability, and the better the colloidal properties due to electrostatic repulsion, and the higher the dispersity. The ζ-potential of SW-AgNPs is negative, suggesting that negatively charged functional groups from the plant extract contribute to the colloidal stability of the AgNPs.

### Bioactivity of SW-AgNPs

#### Effect of SW-AgNPs on plant calli cells

Fresh, friable callus of groundnut variety, TMV2, was inoculated on Murashige and Skoog (MS) solid medium supplemented with 1 mg/L 2,4-Dichlorophenoxyacetic acid (2, 4-D) (MS1) and containing SW-AgNPs in a range of concentrations. The controls included (1) Control-MS medium with 1 mg/L 2, 4-D (MS1), (2) MS medium with 1 mg/L 2, 4-D and (MS1) supplemented with 7.7 ppm plant extract (reductant). The callus was incubated for a period of 15 days in a growth chamber. The effects of the SW-AgNP treatments on the relative visual growth of calli, calli fresh weight, cell death, H_2_O_2_ accumulation and cellular defense/antioxidative enzyme activity were then monitored (Table 2).

**Table 2:** Effect of SW-AgNPs on cell proliferation, and stress related biochemical parameters in groundnut, TMV2, calli.

Tr. No.	Treatments	Fresh weight of calli (mg)	Cell mortality	H_2_O_2_ accumulation	POX activity in calli (μg/min/mg protein)	SOD activity in calli (μg protein for 50 per cent inhibition)
T1	Control -MS medium+2,4-D (MS1)	41.10f	0.98d	1.53f	127.08e	58.03a
T2	MS1+7.7 ppm Sandalwood aqueous leaf extract (reductant)	42.93f	1.05d	1.70f	126.30e	59.83a
T3	MS1+0.5 ppm SW-AgNPs	64.18e	1.18d	1.90ef	133.85de	50.58b
T4	MS1+2 ppm SW-AgNPs	82.03d	1.25d	2.30e	140.98cd	46.73b
T5	MS1+4 ppm SW-AgNPs	100.35c	1.25d	2.88d	144.93c	36.13c
T6	MS1+6 ppm SW-AgNPs	120.48c	1.85c	3.53c	147.25c	30.88d
T7	MS1+8 ppm SW-AgNPs	145.60a	2.60b	4.24b	156.98b	27.35d
T8	MS1+10 ppm SW-AgNPs	117.88b	3.63a	4.94a	169.50a	26.08d
	CD @ 1%	15.58	0.49	0.51	7.82	5.15
	SEM	5.31	0.17	0.17	2.66	1.75
	CV	11.89	19.42	12.00	3.72	8.36

SW-AgNPs-sandalwood aqueous leaf extract derived silver nanoparticles; MS1- MS medium+2,4-D

**Fig 9:**
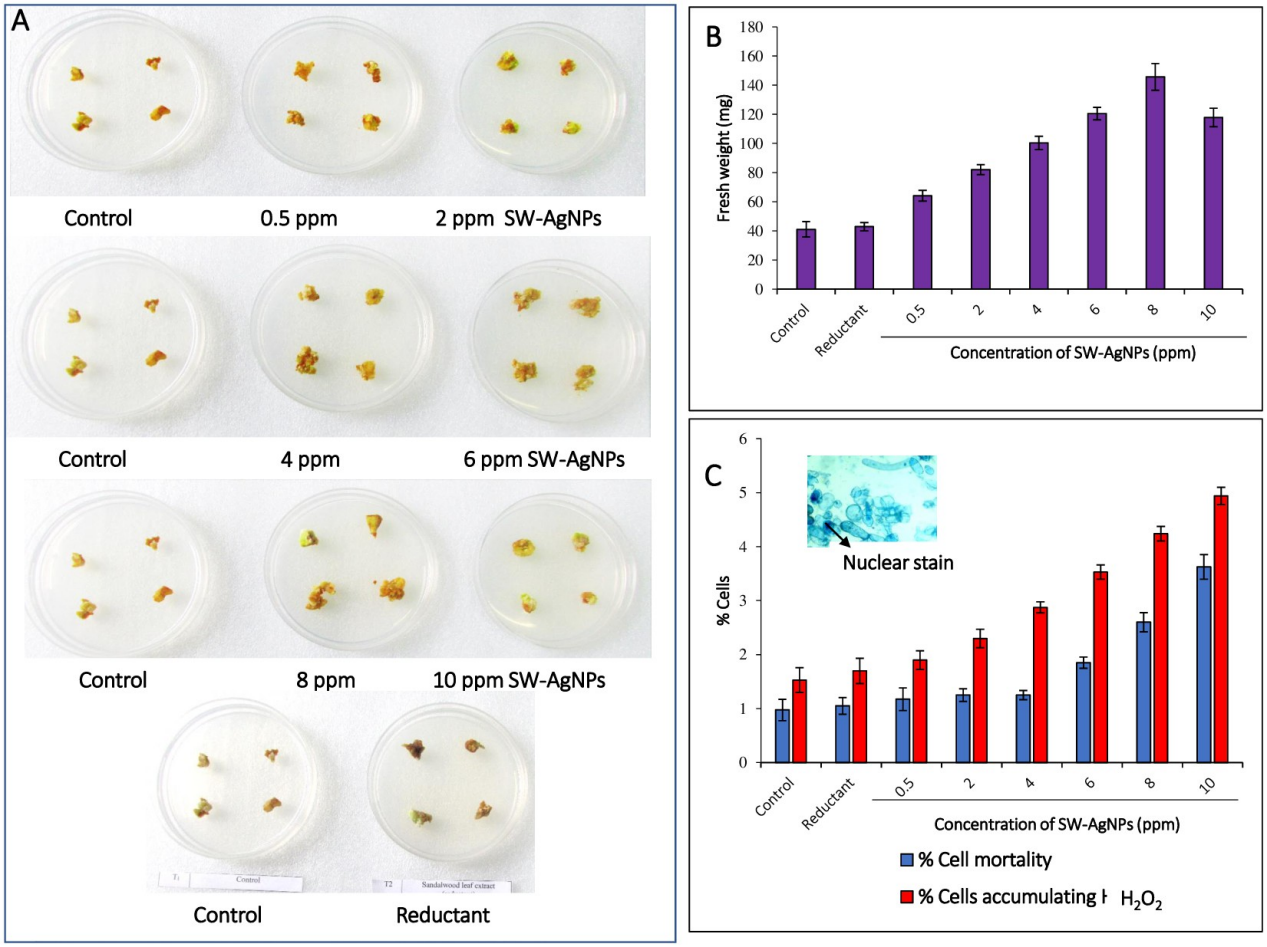
Effect of SW-AgNPs on callus cells: A, Callus of groundnut variety TMV2 subjected to SW-AgNP treatments; B, Fresh weight of callus in different treatments; C, graph depicting per cent mortality (trypan stained cells as Blue bars) and per cent cells accumulating H_2_O_2_ (DAB stained cells as red bar); C inset of micrograph of calli cells stained with Trypan blue.

SW-AgNP treatments resulted in enhanced calli cell proliferation, reflected as increase in calli mass which was visually larger compared to the calli grown in control plates (Fig 9A, Table 2). Fresh weight of the groundnut calli increased in a dose dependent manner up to a concentration of 8 ppm SW-AgNPs, but the trend changed at 10 ppm SW-AgNP with a slight decrease in fresh weight, yet significantly higher than the control (Fig 9B). Highest mean fresh weight of calli was produced at 8 ppm SW-AgNPs (145.60 mg), followed by 6 ppm (120.48 mg), 10 ppm (117.88 mg), 4 ppm (100.35 mg), 2 ppm (82.03 mg), 0.5 ppm of SW-AgNPs (64.18 mg) which were all significantly higher than the control (41.10 mg) and leaf extract-reductant control (42.93 mg) at 1% level of significance. From these results, it is clear that the sandalwood leaf extract in itself did not enhance calli cell proliferation, but SW-AgNPs had a dose dependent positive effect of calli cell proliferation.

The results corroborate with report of Ali *et al*. (2019) [61] that AgNPs effect callus proliferation of *Caralluma tuberculate*, with lower concentration promoting callus proliferation but higher concentration slightly inhibited the growth and proliferation of calli. Ewais *et al*. (2015) [62] observed that addition of AgNPs to MS media results in better proliferation of callus and increased biomass in *Solanum nigrum* L. Begum *et al*., 2020 [63] report that biosynthesized silver nanoparticles show better biomass accumulation and secondary metabolism in callus cultures of *Fagonia indica*, as compared to chemically synthesized AgNPs.

The SW-AgNP induced callus cell proliferation was also observed by us earlier in two other genotypes of groundnut and two varieties of potato (Anil et al, Data unpublished), indicating that the phenomenon of AgNPs enhancing plant calli cell proliferation may be universal across genotypes and plant species. The enhanced cell proliferation resulting from supplementing MS medium with SW-AgNPs suggests that they can be exploited in plant tissue culture protocols for improved plant calli production.

#### Effect of SW-AgNPs on cell mortality of groundnut calli

To evaluate SW-AgNP effect on cell viability, the calli were stained with the vital stain trypan blue to visualize dead cells that show nuclear staining. A representative micrograph is shown in Fig 9C (inset) showing nuclear trypan blue staining. Percentage Cell death was found to increase negligibly in a dose dependent manner in the groundnut calli when exposed to SW-AgNPs (Fig 9C, Table 2). Highest per cent cell death was observed in calli grown in a medium containing 10 ppm SW-AgNPs (3.63%), followed by 8 ppm (2.60%), 6 ppm (1.85%), 4 ppm (1.25%), 2 ppm (1.25%), 0.5 ppm SW-AgNPs (0.66%), reductant control (1.05%), control (0.98%). The cell death is thus low, and below 4%; in other words, Cell viability remained above 96%. The observed cell death can be considered negligible, as in the SW-AgNP treated calli, cell proliferation is radically higher than the controls with significantly higher biomass of calli accumulating during the incubation period.

Thus the observations suggest that although SW-AgNPs have a generalized dose dependent effect on cell death of plant calli, it is marginally higher than controls, and the overall percentage viability remains high, with visible fresh calli, indicating healthy proliferating cells.

#### SW-AgNP induced accumulation of H_2_O_2_ in calli cell

Using a potato calli-in vitro model system, we have earlier demonstrated that pathogen induced accumulation of H_2_O_2_ in calli cells leads to cell death [64]. Although this is a very harmful outcome on calli cells, this phenomenon was surprisingly observed prominently in calli of resistant genotypes only. Indeed, such a response at the whole plant level, actually benefits the plant in the form of hypersensitive response-wherein H_2_O_2_ production at the site of infection kills the cells and curtails spread of disease a very effective mechanism of resistance in plants. Thus H_2_O_2_ induced cell death in calli can be a marker for higher resistance to pathogen at the whole plant level [64]. In the current study, to determine whether the SW-AgNP induced cell death is linked to increased generation of H_2_O_2_, we have monitored the accumulation of H_2_O_2_ in calli cells.

The calli of groundnut, exposed to SW-AgNPs were subjected to Diaminobenzidine (DAB) staining to determine the per cent cells accumulating H_2_O_2_. The per cent cells accumulating H_2_O_2_ increased in a dose dependent manner in SW-AgNP treatments (Fig 9C and Table 2). Highest per cent of cells accumulating H_2_O_2_ was with treatment of 10 ppm SW-AgNPs (4.94%), followed by 8 ppm (4.24%), 6 ppm (3.53%), 4 ppm (2.88%), 2 ppm (2.30%), 0.5 ppm SW-AgNPs (1.90%), reductant-control (1.70%) and control (1.53%). Although this is a minor increase, it is a significant one. The higher per cent cells accumulating H_2_O_2_ can attribute to the marginally higher cell death observed in the SW-AgNP treatments. Increase in H_2_O_2_ by SW-AgNP exposure could mimic a hypersensitive response, and may enhance pathogen resistance in crops exposed to AgNPs, and this hypothesis warrants further studies.

#### SW-AgNP induced changes in peroxidase (POX) and superoxide dismutase (SOD) activities in calli cells

**Fig 10:**
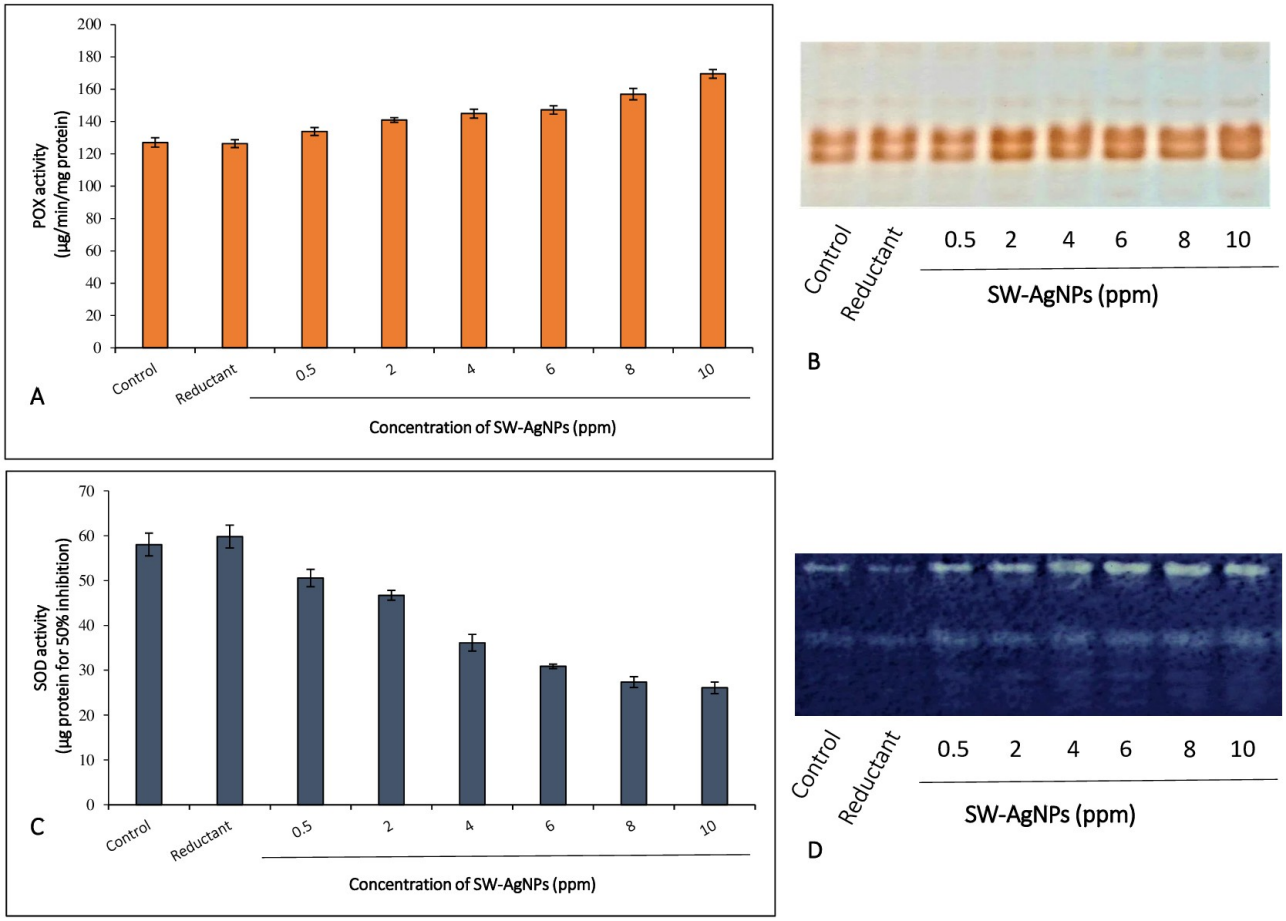
Effect of SW-AgNPs on defense/stress enzymes superoxide dismutase and peroxidase activities in the protein extracts of groundnut TMV-2 calli: A, peroxidase activity; B, In gel POX isozyme activity; C, SOD activity; D, In gel SOD isozyme activity; Reductant, aqueous sandalwood leaf extract.

As the DAB staining indicates an increase in accumulation of H_2_O_2_ in calli cells, it can be inferred that there should be a concomitant increase in antioxidant enzymes such as SOD and Peroxidase. These antioxidant enzymes play significant roles in plant defense and stress responses. Peroxidase is widely distributed in all higher plants and protects cells against the destructive influence of H_2_O_2_ by catalyzing its decomposition through the oxidation of phenolic compounds. Indeed, with increasing concentration of SW-AgNPs in the media, enhanced activities of peroxidases and superoxide dismutase were observed (Fig 10, Table 2). The highest peroxidase activity was observed at 10 ppm SW-AgNPs (169.50 μg/min/mg protein), followed by 8 ppm (156.98 μg/min/mg protein), 6 ppm (147.25 μg/min/mg protein), 4 ppm (144.93 μg/min/mg protein), 2 ppm (140.98 μg/min/mg protein), 0.5 ppm SW-AgNPs (133.85 μg/min/mg protein). All SW-AgNP treatments exhibited significantly higher peroxidase activity than that of control (127.08 μg/min/mg protein) and reductant (126.30 μg/min/mg protein) at 1 per cent level of significance (Fig 10A, Table 2).

Peroxidase isozyme activity (POX) was also analysed by an in-gel assay which showed that peroxidase activity clearly increased when calli were exposed to SW-AgNPs (Fig 10B). This increased POX activity may result in increased ROS scavenging and thus an effective mechanism to cope up with the stress posed by AgNP exposure. This study did record higher per cent of cells showing peroxide accumulation (Fig 9C, Table 2) as compared to control. However, the per cent of cells accumulating peroxide even in the highest concentration of SW-AgNPs used was only 4.94%, with an increase of only $\tilde{2}\%$ over the controls (Table 2) which is quite negligible when the entire population of cells is considered. It is possible that a robust POX activity scavenges the peroxide, keeping levels low in the calli, even with exposure to SW-AgNPs, thus resulting in the low mortality observed.

Superoxide dismutase (SOD) is a pivotal enzyme in ROS scavenging mechanisms and converts the highly cytotoxic superoxide anion to H_2_O_2_. The higher H_2_O_2_ levels detected in SW-AgNPs exposed calli cells suggested higher activity of SOD. Indeed, the spectroscopic assay for SOD clearly showed enhanced SOD activity with SW-AgNP exposure in a dose dependent manner (Table 2, Fig 10C) The highest superoxide dismutase activity was observed at 10 ppm SW-AgNPs treatment (26.08 μg protein for 50% inhibition), followed by 8 ppm (27.35 μg protein for 50% inhibition), 6 ppm (30.88 μg protein for 50% inhibition), 4 ppm (36.13 μg protein for 50% inhibition), 2 ppm (46.73 μg protein for 50% inhibition), 0.5 ppm (50.58 μg protein for 50% inhibition) of SW-AgNPs which were significantly higher than that of control (58.03 μg protein for 50% inhibition) and reductant (59.83 μg protein for 50% inhibition) at 1% level of significance (Table 2, Fig 10C).

In gel assays also showed a clear increase in SOD activity in calli when exposed to SW-AgNPs, (Fig 10D). SOD activity, H_2_O_2_ and POX activity have a pivotal role in defense and abiotic stress tolerance mechanisms in plants [65–67]. Higher activities of SOD and peroxidases with exposure to SW-AgNPs can result in effective scavenging of harmful superoxide, and H_2_O_2_.

The current investigation thus demonstrates that exposure of plant calli cells to SW-AgNPs has no adverse effect on calli growth, in fact it enhances it considerably, and viability tests show more than 96% viability in cells exposed to SW-AgNPs. In fact the treatment induces the calli to proliferate as evidenced by the fresh friable calli on the surface of each callus clump on the media. The cells show induction of SOD and peroxidase enzyme activities and moderate accumulation of peroxide. The results thus suggest that although the exposure to SW-AgNPs is a mild abiotic stress to plant cells, the cells are able to respond in an appropriate manner in terms of production of ROS and effective scavenging of peroxide so that cells survive and proliferate with the exposure to SW-AgNPs. This is a unique insight that assures the safe use of SW-AgNPs on plants and points at the potential of application of SW-AgNPs against pathogens and pests or even as a growth promoter of crop plants. To our knowledge this is the first study to evaluate the plant cellular response to AgNPs. The increase of peroxide and significant increase in SOD and peroxidase activities may indeed be additional benefits to plants as these are components of the plants defense and abiotic stress response pathways and may induce the defense preparedness and abiotic stress tolerance of the crop in the field. Further testing of this hypothesis is warranted.

#### Effect of SW-AgNPs on calli -secreted biomolecules

**Fig 11:**
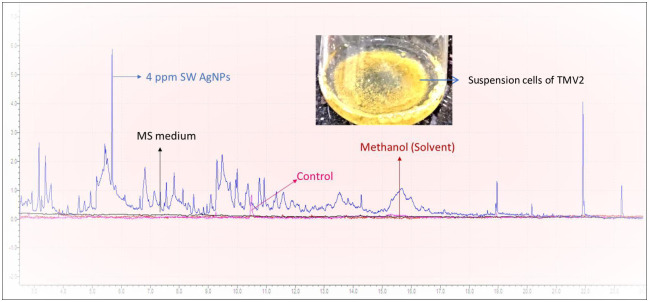
GC-MS generated chromatogram of methanolic extracts of secreted biomolecules from suspension cells of groundnut, variety TMV2: Blue chromatogram indicates GC-peaks of secreted molecules from suspension cells exposed to 4 ppm SW-AgNPs; pink chromatogram represents GC-peaks secreted molecules from control suspension cells; light pink chromatogram represents the solvent methanol alone and the black chromatogram represents methanolic extract of MS media alone. Methanol and MS media extract serve as controls. Inset, photo of suspension cells of TMV2 (groundnut) in flask containing liquid MS medium, supplemented with 1 mg L^-1^ 2, 4-D and 4 ppm SW-AgNPs.

GC-MS analysis of methanolic extract of the calli suspension medium showed that exposure of calli suspension cells to SW-AgNPs induced the secretion of several unique compounds as compared to control (unexposed cells) (Fig 11).

Some of the unique compounds secreted by groundnut calli when exposed to SW-AgNPs are 2,2-Bioxirane, 6,7-Dihydropyrido(2,3-d)pyridazine-5,8-dione, dl-Threitol, Crotonyl isothiocyanate, Butyrolactone, 2-Hydroxy-gamma-butyrolactone, N-Isopropylcyclopropanecarboxamide, Guanidineacetic acid, Maltol, Furandimethanol, Ethanamine, 2-propoxy, 3-Amino-2-oxazolidinone. α-Methylene butyrolactone, is a fungitoxic substance present in the white skin of tulip bulbs which prevents the entry of *Fusarium oxysporum* into the bulb [68]. 1,4-dithio-DL-threitol compound similar to dl-Threitol was found to improve the callus formation, shoot differentiation and growth, and shoot rooting by inhibiting tissue necrosis during the initiation of cultures and subculture of shoots of Virginia pine [69]. Isothiocyanates are well known for their anti-herbivore properties. Maltol is an important component of a direct defense strategy in balsam fir against spruce budworm herbivory [70]. Oxazolidinones is known to have antimicrobial properties against gram positive bacteria [71]. The presence of a DL-threitol may be one of the key reasons why AgNPs induce calli cell proliferation observed in this investigation, and in our unpublished earlier work and in other reports. The secretion of anti-fungal and anti-microbial biomolecules is interesting and calli cultures can be exploited to produce such compounds that can have application in agriculture. The application of nanoparticles in Agriculture is still at its infancy, this study provides evidence that SW-AgNPs are not cytotoxic to plant cells, and in fact that they enhance calli cell proliferation, enhance an antioxidant response and induce the synthesis of several useful plant metabolites that would help in promoting growth, and mitigating biotic and abiotic stresses. The results overarchingly point to the safe and beneficially application of SW-AgNPs in agriculture.

#### Cytotoxic effect of SW-AgNPs on cervical cancer cell lines

**Fig 12:**
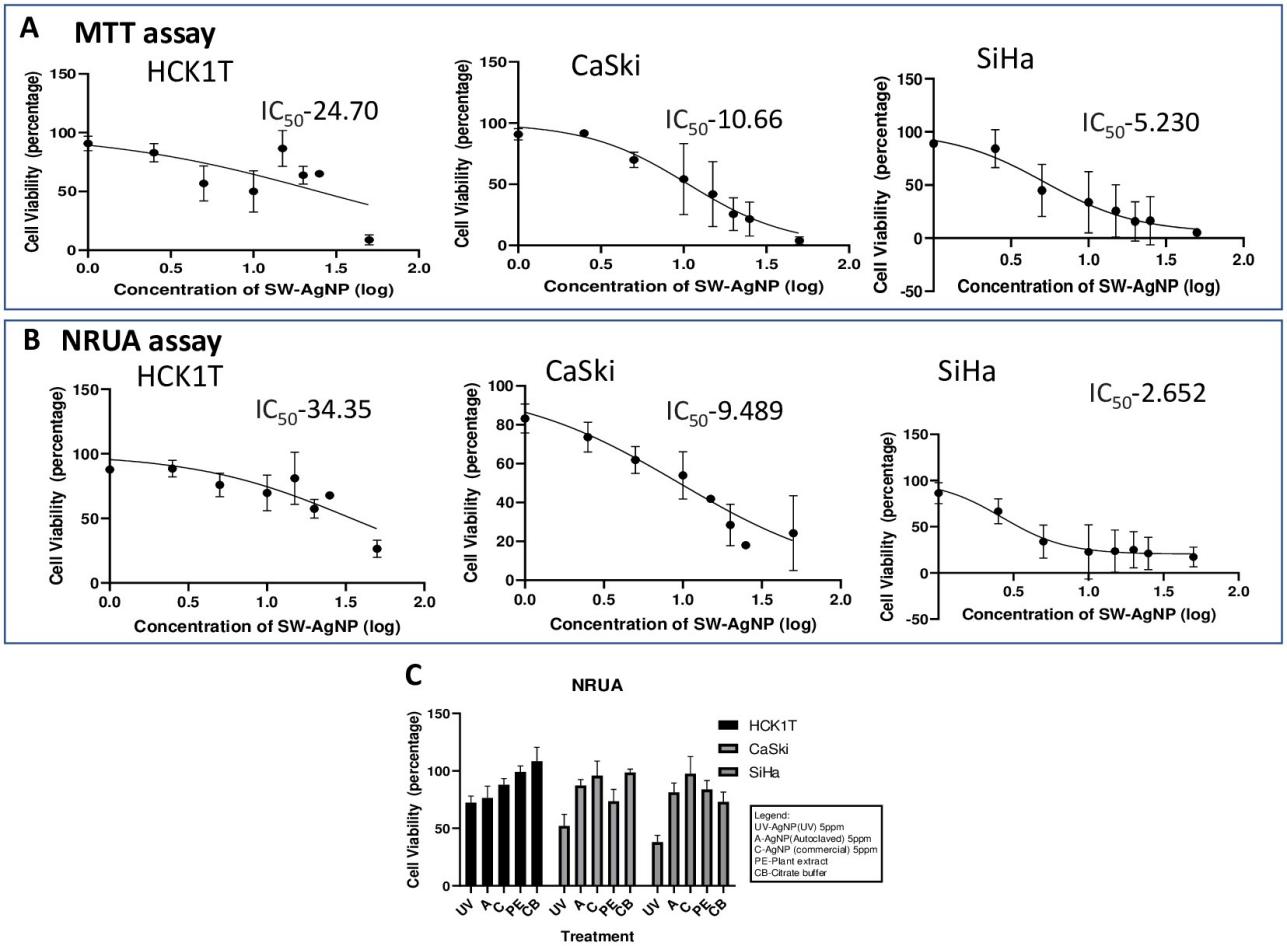
Cytotoxicity of SW-AgNPs on cervical cell lines: A, cytotoxicity evaluated using MTT assay; B, Cytotoxicty as evaluated by the NRU assay. CaSKi and SiHA are cervical cancer cell lines and HCK1T is a normal cervical cell line. C, Cytotoxicity of UV sterilized SW-AgNPs (UV) on cervical cell lines as compared to autoclaved SW-AgNPs (A), commercial AgNP (C), Plant Extract (PE) and citrate buffer (CB).

Ag nanoparticles have been reported to exhibit anticancer properties [23]. Do SW-AgNPs also have inhibitory effect on cancer cells or would they mimic the effect they showed on plant calli depicted as cell proliferative action at low concentrations? To obtain some insight to this question, cervical cancer cell lines (CaSki and SiHa) and a normal control cervical cell line HCK1T were exposed to a range of concentrations of SW-AgNPs, followed by assessment of cytotoxicity using MTT and Neutral Red Uptake (NRU) assays (Fig 12A & 12B). Both MTT and NRU assays showed that SW-AgNPs had a dose dependent toxic response towards the cancer cell lines with very low concentrations causing cytotoxicity in cells (CaSki and SiHa). Comparatively, the effect of SW-AgNPs on normal cervical cell line HCK1T, was not dose dependent and cells remained almost unaffected at lower concentrations, and cytotoxicity was observed only at higher concentrations of SW-AgNP. The IC_50_ (50% cell death) of the cervical cancer cell line CaSki was found to be 10.66 ppm of UV sterilized SW-AgNPs from the MTT assay and 9.48 ppm from NRU assays, SiHa was found to be 5.23 ppm of UV sterilized SW-AgNPs from the MTT assay and 2.65 ppm from NRU assay. On the other hand, IC_50_ of the normal cervical cell line, HCK1T was clearly higher when compared to the cancer cell lines at 24.70 ppm of UV sterilized SW-AgNPs from the MTT assay and 34.35 ppm from neutral red uptake assay.

The cytotoxic effect of SW- AgNPs may be due to their antineoplastic nature and their capacity via various molecular mechanisms to induce cell death. It is important to note that the SW-AgNPs used in this study were much more effective in inducing death in cancer cell lines than the commercial AgNP control (Fig 12C). The cell viability was observed in a number of controls including citrate buffer, capping material (plant extract), commercial AgNP and autoclaved SW-AgNP to understand the component instrumental in contributing to selective cell death in cervical cancer cells. The SW-AgNP that was UV sterilized was able to effectively and selectively kill the cervical cancer cells, with comparatively lower cytotoxic effect by autoclaved SW-AgNP, suggesting that at least some of the plant derived capping material was heat sensitive. These inhibitory effects were not observed in cervical cells treated with the various controls, including the commercial chemically synthesized AgNP, as seen in the graph (Fig 12C). It is possible that the capping material from the plant extract contributes to the effectiveness of the in-house synthesized SW-AgNPs.

Similar studies with green AgNPs synthesis by using *Azadirachta indica* leaf extract had a cytotoxic effect on cervical cancer cell line Siha with an IC_50_ of ≤4.25 μg/mL [72], which is comparable to IC_50_ values obtained for SW-AgNPs against cervical cancer cell lines in this study. Sukirtha *et al*. (2012) [73], used spherical shaped AgNPs of 78 nm size synthesized using *Melia azedarach* leaf extract to demonstrate cytotoxic effect on HeLa with inhibitory action at 300 μg/mL. Similar results were also obtained by other research groups [74, 75] in HeLa cells when they were exposed to AgNPs. Compared to these earlier reports SW-AgNPs showed $\tilde{1}0$ fold lower IC_50_ values, indicating greater effectiveness against cervical cancer cell lines. Abdellatif *et al* (2022) [76] demonstrate that green synthesized AgNPs reduced using *Allium cepa* extracts inhibited colorectal cancer cell lines and induced apoptosis in these cells.

#### DNA damage analysis

**Fig 13:**
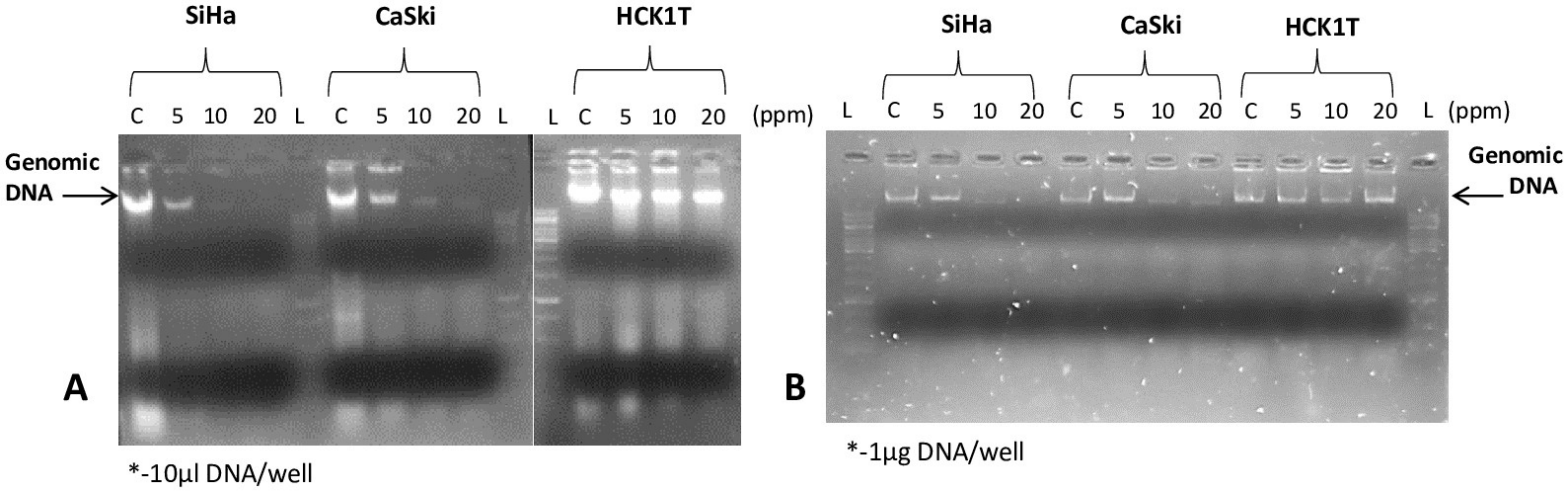
DNA damage assay: Total DNA was isolated from the cervical cell lines and then evaluated by agarose gel electrophoresis. A, 10 μl of DNA from the SW-AgNP treated cervical lines were loaded per well; B, The DNA was estimated and 1 μg DNA of the cervical cell lines was loaded per well. L- DNA marker, C- Untreated Control, 5, 10, 20- concentration of UV treated SW-AgNP in ppm.

DNA damage in the cell lines exposed to SW-AgNPs (5–20 ppm) was evaluated to assess cytotoxic effect of the treatment. This assay is a qualitative method of analysing cell viability. The process of DNA isolation involves cell lysis through TES buffer and enzymatic digestion. Hence, there is no phase separation step involved in the process of genomic DNA isolation therefore the damaged DNA was not removed in any of the steps therefore minimizing DNA loss. Results showed that there was a clear reduction in the DNA concentration of the SW-AgNPs treated cells. DNA concentration in the cancerous cell lines CaSki was 639 ng/μl and 174.7 ng/μl in control versus 10 ppm SW-AgNPs treated respectively. DNA concentrations in SiHa cells was 792.6 ng/μl and 80.7 ng/μl in control versus 10 ppm SW-AgNPs treated respectively. The SW-AgNP treatments reduced DNA levels greatly in cancer cell lines as compared to the normal cell line, HCK1T, that showed DNA concentrations of 1542.4 ng/μl, 1187 ng/μl in control versus10 ppm SW-AgNPs treated respectively. The SW-AgNP treatment-induced reduction in DNA was observed even in the agarose gel (Fig 13), where sample wells were loaded with equal amount of DNA (1 μg). The DNA band can be seen clearly in the control lane of all cell lines showing minimal damage. But in the SW-AgNPs (5–20 ppm) treated cancer cells, DNA bands are very faint due to loss of intact DNA upon damage. In the case of HCK1T (normal cervical cell line), DNA is observed to be relatively intact even upon treatment with 20 ppm SW-AgNPs (Fig 13B).

In terms of the DNA content, the cervical cancer cells treated with SW-AgNPs was seen to possess significantly lower amounts when compared to its untreated counterpart. While, the normal HCK1T cervical cells showed relatively low amounts of DNA damage under the SW-AgNPs treated and untreated conditions that was reflected in the minimal difference in DNA content (Fig 13A). This could be attributed to SW-AgNPs being selectively lethal to the cancer cells as seen with lower IC_50_ values in assays for cytotoxicity and cell viability in cancer cell lines that also was reflected in the SW-AgNP treated DNA damage.

#### Morphological analysis of cervical cell lines

**Fig 14:**
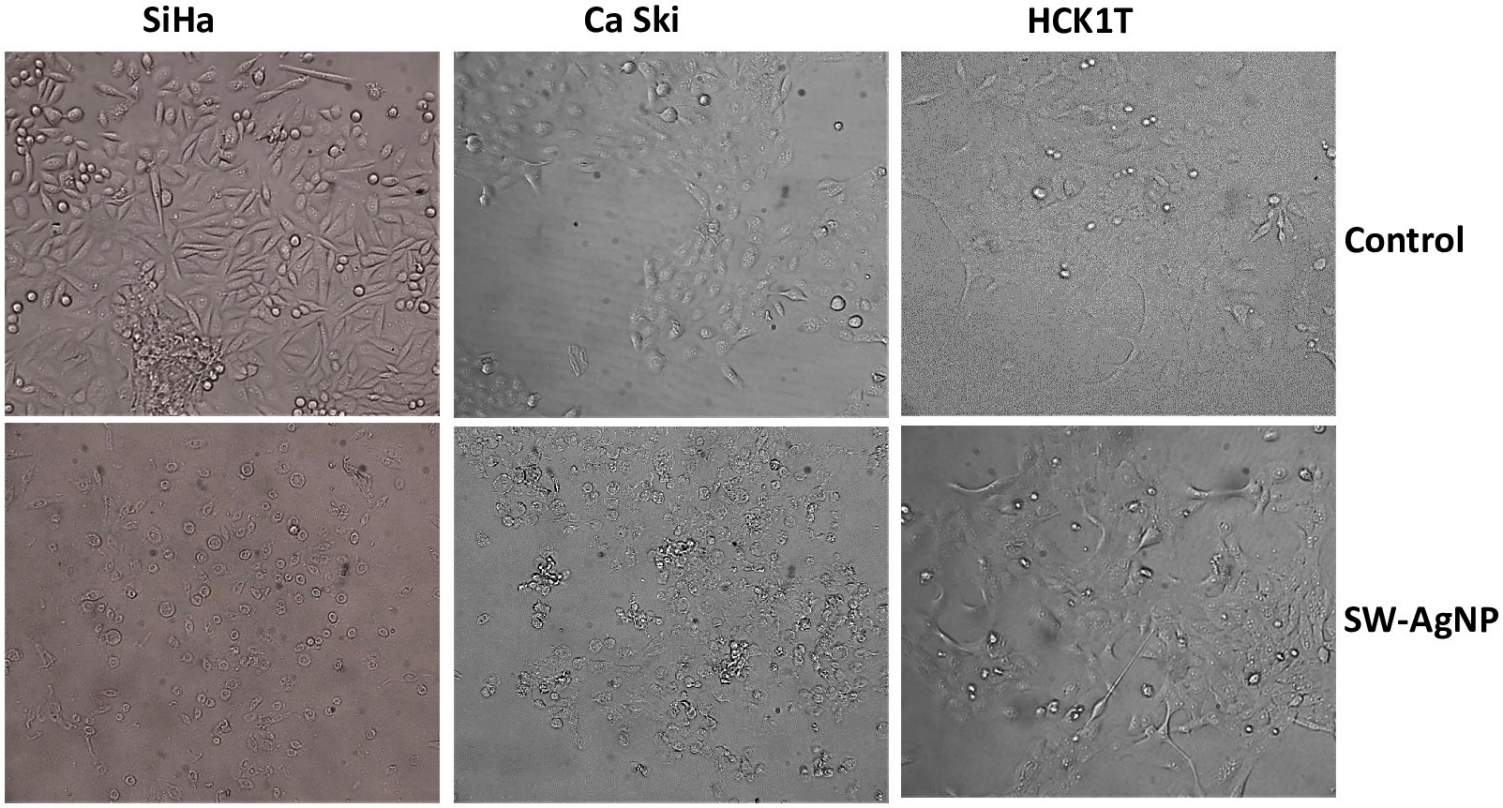
Microscopic view of cervical cancer and normal cell lines. A; media control, B; 20 ppm UV sterilized SW-AgNPs.

Morphologically, SW-AgNPs treated cancer cells showed significant change in their shape with many cells having shrunken appearance, loss of cell adhesion capacity (Fig 14). However, control cells retained their normal morphology and continued to grow well by attaching to the surface of the culture dish. In case of normal cell line HCK1T, SW-AgNPs treatment did not affected the morphology as seen in the cancer cell lines (SiHa, CaSki). Similar cytotoxic effect and morphological abnormalities (cell shrinkage, membrane destruction, chromatin condensation, protrusion of microspikes and fragmentation of nuclei) were reported with AgNPs synthesized using *Perilla frutescens* extract in cancer cell lines COLO205 and LNCaP [13]. Phytosynthesized AgNPs using *Mentha arvensis* exhibited anticancer activity in colon cancer cell lines via apoptosis with cell membrane fragmentation, and cell cycle suspension [77]. Phytosynthesized AgNPs using *Clinacanthus nutans* also lead to cytotoxicity in oral squamous cancer cells with membrane blebbing, nuclear fragmentation, and chromatin condensation [78]. The current study also reports clear morphological changes in cervical cancer cells with significant DNA damage. The reason for SW-AgNP induced cytotoxicity to cancer cells, can potentially be attributed to DNA damage leading to and cell death. Further investigation is necessary to understand the mechanism of action triggering cell death.

The results obtained from this study show that SW-AgNPs have higher cytotoxic effect towards the cancer cells compared to the normal counterpart, suggesting that these SW-AgNPs have potential to be used in cancer therapy. The role of Alpha Santalol from sandalwood oil has been studied extensively in the context of cancer for its anticancer properties, but the source of the oil come from the heartwood of the tree that requires years to grow. Instead, the leaves from the plant can be easily harvested as the starting material for the phytosynthesis of the AgNPs and has also demonstrated effective anticancer properties.

The application of nanoparticles in Agriculture is still at its infancy, this study provides evidence that SW-AgNPs are not cytotoxic to plant cells, and in fact that they enhances calli cell proliferation and induce the synthesis of several useful plant metabolites. The observations suggest that the application of AgNPs to crops can be safe and beneficial. The cytotoxicity of phytosynthesized SW-AgNPs on cancer cells is significant as compared to commercial AgNPs, indicating that the capping with plant metabolites have additional beneficial effects, and can be preferred over chemically synthesized AgNPs in applications in medicine. The sustainability, economic feasibility, and environmental compatibility of the proposed phytosynthesis procedure could provide a broad range of uses for the resulting silver nanoparticles.

## Conclusion

The study biogenerates fine quality silver nanoparticles (SW-AgNPs) using an aqueous sandalwood leaf extract at ambient temperature, with minimal stirring, making this an efficient, economical and eco-friendly method of biosynthesis. The FTIR analysis indicates that the bioreduction of Ag^+^ to Ag^o^ and capping of the resulting SW-AgNPs are facilitated by proteins, aromatic polyphenols, flavonoids and lipids. SEM, AFM and TEM analysis demonstrates that SW-AgNPs are spherical, with an estimated size range of 10–32 nm. DLS indicates a hydrodynamic average particle size of 49.53 nm with a predominant negative *ζ*-potential (peaks at—9.32 mV and -41.1 mV). This implicates negatively charged functional groups derived from the plant extract in the colloidal stability of the SW-AgNPs. SEM based EDAX and XRD analysis clearly demonstrate the formation of silver nanoparticles, and point to a FCC crystalline structure. Further, the study examines the effects of SW-AgNPs on plant calli cells and on cervical cell lines. In case of plant calli, SW-AgNPs boosts cell proliferation, induces antioxidant response and the secretion of metabolites that are implicated in antimicrobial and plant cell proliferative functions. SW-AgNPs enhanced the callus by over 70% compared to the control. In case of cervical cancer cell lines, SW-AgNPs were specifically and significantly cytotoxic. Cervical cancer cells, CaSki (IC_50_–10.66 and 9.49 ppm respectively by MTT and NRU assays) and SiHa (IC_50_–5.23 ppm and 2.65 ppm respectively by MTT and NRU assays) showed cell death at lower concentration of SW-AgNPs compared to normal cervical cells HCK1T (IC_50_-24.70 ppm and 34.35 ppm respectively by MTT and NRU assays). In addition, the inhibition was much higher with SW-AgNPs than with chemically synthesized commercial AgNPs or with autoclaved SW-AgNPs indicating a superior bioactivity, and the contribution of heat labile phytochemicals (capping) in the bioactivity. This demonstrates that silver nanoparticles (AgNPs) synthesized from aqueous sandalwood extract has the ability to target the cancerous cells selectively and effectively. Overall the findings point to the potential application of SW-AgNPs in plant cell culture, agriculture, and in cancer therapy. Further identification of the active molecules involved in capping of SW-AgNPs and studies on the potential applications in cancer therapy and in agriculture are warranted.

## Materials and methods

### Chemicals

Most of the utilized chemicals in this study were obtained from Merck and used as received without any further purification. Commercial silver nanoparticle and chemicals used for the cervical cell line experiments were procured from Sigma-Aldrich.

### Screening of plant species for levels of phenolic and flavonoids

Secondary metabolites can act as reducing agents for the reduction of silver ion to silver nanoparticle. In this context, the study was initiated by screening several plant species for their total phenolic and flavonoid content. Easily available candidate medicinal plants and tree species such as Neem (*Azadirachta indica*), Amrutha balli (*Tinospora cordifolia*), Sandalwood (*Santalum album* L.), Brahmi (*Bacopa monnieri* L., Pongemia (*Pongamia* *pinnata* L.) and tamarind (*Tamarindus indica*) were screened for leaf total phenolic and flavonoid levels.

### Phenolic and flavonoid extraction

One gram of leaf sample was homogenized in 10 ml of 80% ethanol in a pestle and mortar. The homogenate was centrifuged at 10,000 rpm for 20 min, the supernatant was collected and the residue re-extracted with five times the volume of 80% ethanol and re centrifuged. After this the supernatant was collected and evaporated to dryness and the residue dissolved in 2 ml of distilled water.

### Estimation of total phenolics

Phenols react with an oxidizing agent phosphomolybdate in Folin- Ciocalteu reagent (FCR) under alkaline conditions which results in the formation of a blue coloured complex which was measured at 650 nm spectrophotometrically as per the method of Bray and Thrope, 1954 [79] with slight modifications. In brief, the ethanolic extract (0.2 ml) was diluted with 3 ml distilled water followed by the addition of 0.5 ml Folin- ciocalteau reagent. The contents were mixed thoroughly. After 3 min, 2 ml of saturated sodium carbonate was added and the contents were allowed to stand for 1 min in boiling water bath, cooled and absorbance was measured at 650 nm(using Cary 300 UV-VIS spectroscope model by Varian spectrometer). The phenolic concentration present in the test samples were calculated using standard curve of gallic acid and the concentration was expressed as mg gallic acid equivalents (GAE).

### Estimation of flavonoids

Total flavonoid content was measured by the aluminium chloride colorimetric assay [80]. The ethanolic extract (0.2 ml) was diluted with distilled water to make the volume to 1 ml and then 1.8 ml 95% methanol, 0.1 ml of 1 M sodium acetate and 0.1 ml 10% aluminium chloride was added to the same. The absorbance was recorded at 415 nm using spectrophotometer against the blank. The flavonoid concentration present in the test examples were calculated using standard curve of Rutin and the concentration is expressed as mg Rutin equivalents (RE).

#### Phytosynthesis of silver nanoparticles

Healthy and mature leaves of *Santalum album* L. were collected from the University of Agricultural Sciences, GKVK campus, Bangalore, India. Initially, the *S album* leaves were washed thoroughly with tap water and then rinsed with distilled water. After which, the leaves were cleaned with double distilled water and then surface dried by using blotting paper. For preparing the aqueous leaf extract, 1 g of fresh leaves was homogenized and suspended in 10 ml double distilled water. The homogenized sample was centrifuged at 10,000 rpm for 15 min. Following which, the aqueous supernatant was collected for use as the reductant in the phytosynthesis procedure.

The aqueous leaf extract was used for the reduction of Ag^+^ ions to Ag^o^. The silver nitrate (AgNO_3_) was purchased from Sigma-Aldrich chemicals, USA. Aqueous solution of 1 mM AgNO_3_ was prepared using distilled water. Leaf extract (2 mL) was added to 8 mL of 1 mM aqueous AgNO_3_ solution and mixed for a minute. The pH of the reaction mixture was estimated to be 6.8–7.2. The flasks containing the mixture were incubated at room temperature or at higher temperature (95°C) over a period of 8 hrs for the formation of silver nanoparticles. The phytosynthesized AgNPs using aqueous sandalwood leaf extract will be referred to as SW-AgNPs henceforth.

#### Characterization of the phytosynthesized Silver Nanoparticles

The nanoparticles were characterized by UV-Vis spectroscopy, FTIR (Fourier transform infra-red spectroscopy) analysis SEM (Scanning Electron Microscopy) analysis, AFM (Atomic force microscopy) and XRD (X-ray diffraction), TEM (Transmission Electron microscopy) and DLS (Dynamic Light scattering).

#### UV spectrophotometric analysis

The bioreduction of silver ions at room temperature and 95°C using sandalwood leaf extract was monitored by UV -Visible spectral measurements using a Cary 300 UV-VIS spectroscope model by Varian spectrometer. UV–Visible spectra across 200–800 nm wavelength range operated at a resolution of 1 nm of aliquots from the phytosynthesis mixture were monitored as a function of reaction time. Similarly, spectra of controls- 1 mM AgNO_3_, water and plant extract were also recorded.

#### FTIR analysis

FTIR was carried out based on the principle that most molecules absorb light in the infra-red region of the electromagnetic spectrum. This absorption corresponds to the bond vibrations present in the molecule. In this study the frequency range was measured as wave numbers typically over the range 4000—400 cm^-1^ using Thermo scientific Nicolet 6700 spectrophotometer. The FTIR measurements were carried out for sandalwood leaf extract as a control, and for the washed SW-AgNPs (free from leaf extract or biomass residue in the suspension) prepared both at room temperature and at 95°C.

The sample was very finely ground to reduce scattering losses and absorption band distortions. Two stainless steel disks were taken out of the desiccator. A piece of the precut cardboard (in the tin can next to the oven) was placed on top of one disk and the cutout hole was filled with the 3 μl of sample mixture. FTIR is particularly useful for identification of organic molecular groups and compounds due to the range of functional groups, side chains and cross-links involved, all of which will have characteristic vibration frequencies in the infra-red, as per the procedure of Harish Kumar *et al*., (2017) [81].

#### X-Ray Diffraction (XRD) analysis of SW-AgNPs

XRD is a valuable characterization tool to prove the formation of AgNPs, determine the crystal structure and calculate the crystalline nature of nanoparticles [82].

In this study, the nature of biosynthesized SW-AgNPs was determined using XRD set up at 30 kv and 40 mA with Cu Kα radians at an angle of 2. The SW-AgNPs prepared at room temperature and at 95°C were loaded by even spreading on sample holder or glass slides; the sample holder was positioned appropriately in XRD machine and analyzed using the inbuilt software.

#### SEM analysis

SEM measurements were performed on a Merlin Compact VP machine model of Carl Zeiss Company. SW-AgNPs prepared at two variant temperatures (Room temperature or 95°C) were analyzed by SEM. Sample preparation for scanning electron microscopy involved placing 20 μL of the washed nanoparticle suspension on a silica coated slide and later exposed to infrared light for 30 minutes for solvent evaporation. The slide was then observed under a scanning electron microscope. The elemental composition of the synthesized silver nanoparticles was analyzed by Energy dispersive X-ray microanalysis spectroscopy by selecting a small area in the field containing nanoparticles for the analysis. Multiple images were acquired for all samples and later analyzed using the software ImageJ. The size (diameter) of randomly selected 25 nanoparticles from both treatments (RT and 95°C preparations) were measured and mean values attained.

#### Atomic Force Microscopy (AFM)

The size, shape and surface nature of SW- AgNPs were characterized by using AFM. The SW-AgNPs solutions were sonicated for 15 min at room temperature using an ultra-sonicator. The SW-AgNPs solution was later dried to form a thin layer on mica-based glass slide which was used to visualize under the AFM.

#### Transmission electron microscopy

The transmission electron microscopy (TEM) studies were performed with System TEM FEI- Titan-Themis at 300kV. Samples were prepared as collidal solution dropcost on Corbon coated Cu 300 mesh grid and the solvent was evaporated.

#### Particle size, and ζ-potential measurements

The average hydrodynamic particle size distribution, and *ζ* potential (surface charge) of the nanoparticles were measured using a Zetasizer nanoZS (Malvern Instruments, Malvern, U.K.) by the dynamic light scattering (DLS) technique. The size measurements were carried out at 25°C, adjusting the light scattering angle at 90°. The surface charge (*ζ*-potential) of the SW-AgNPs was measured using an electrophoretic cell under an electric field. The sample was prepared by diluting the nanoparticles with MilliQ water to obtain a well dispersed suspension.

### Evaluation of effect of SW-AgNPs on plant calli cells

#### Groundnut (variety-TMV-2) callus induction

Surface sterilized pre-soaked groundnut variety, TMV-2 seeds were inoculated on a plain Murashige and Skoog, (1967) [83] (MS) media (without any growth hormones) and incubated at 22°C. From the newly germinated *in vitro* groundnut seedlings, leaves were collected and used as explant for callus induction in MS media containing 1 mg/L Napthalic acetic acid (NAA) and 1 mg/L Benzyl amino purine (BAP). The callus induced at the cut edges of leaf discs were subcultured into MS medium containing 2 mg/L 2, 4-D for further callus proliferation and subcultured into MS medium containing 1 mg/L 2, 4-D (MS1) for callus proliferation and maintenance.

#### SW-AgNP treatments imposed on groundnut, TMV-2, callus

To evaluate the effect of SW-AgNPs on calli cells, MS Medium was prepared as detailed above, along with supplementation of SW-AgNPs at 0, 0.5, to 10 ppm. MS medium without silver nanoparticles was considered as the control. MS medium containing filter sterilized sandalwood leaf extract (reductant) was also included in the experiment as another control. A packed cell volume of 100 microliters of the calli was inoculated on to the media surface as four replicates equidistant from each other. Three plate replicates were also maintained for each of the treatments. The calli were allowed to proliferate at 22°C in defuse light conditions for 20 days before evaluation of cell viability and the activity of defense/antioxidant enzymes.

#### Trypan blue staining for viability of calli cells

The viability of callus cells was estimated by dye exclusion method using Trypan blue vital stain. The calli of TMV2 groundnut genotype exposed to different concentrations of SW-AgNPs in the medium were tested for per cent cell death. A packed cell volume (PCV) of calli (200 μl) from the different treatments were suspended in liquid MS media in a microfuge tube and placed in rotary shaker for about 10–20 min. After centrifugation at 4000 rpm for 5 min, the supernatant was discarded and 100 μl of 0.4% trypan blue stain was added and placed again in a rotary shaker for 10 min to allow the cells to take up the stain. Then the cells were centrifuged at 4000 rpm for 5 min and the supernatant was discarded. The stained calli cells were washed twice with liquid MS media and centrifuged at 4000 rpm for 5 min to remove the nonspecific traces of trypan blue stain. The mortality of cells was calculated by counting the total number of cells and the number of nuclear stained, dead cells in 4 microscopic fields in three replicate plates as per the procedure followed by Anil et al. 2007 [84].

#### DAB staining for H_2_O_2_ accumulation in calli cells

The production of hydrogen peroxide (H_2_O_2_) in calli cells as a response to exposure to SW-AgNP was detected using 3, 3‘-diaminobenzidine (DAB) staining method described by Orozco- Cardenas and Ryan (1999) [85] with slight modification. DAB precipitates and turns deep brown in the presence of H_2_O_2_.

The calli of groundnut exposed to different concentrations of SW-AgNPs were placed in 10 ml of DAB staining solution (1mg/ml DW) for 2 hours to allow the callus to take up the DAB stain. The callus was then washed with hot ethanol to remove non specific DAB staining, and the cells were observed under a microscope at 10X magnification. The dark brown stained cells indicate intracellular accumulation of H_2_O_2_. The number of cells with dark brown colour and the total number cells were counted in four microscopic fields in a slide with three sample replicates and the per cent cells accumulating H_2_O_2_was calculated.

### Defense/antioxidant enzyme assays

#### Soluble protein extraction from groundnut callus

Groundnut calli from different treatments were frozen in liquid nitrogen to prevent proteolytic activity and homogenized using a pestle and mortar. The homogenate was then suspended (1:10) in extraction buffer [Phosphate buffer 0.1 M of pH 7.8, 1 mM PMSF (protease inhibitor) and 0.1% of polyvinyl pyrolidon (PVP)] and held on ice for 15 min. The crude protein extracts were centrifuged at 14,000 rpm at 4°C for 30 min. The pellet was discarded and the supernatant containing the soluble proteins was stored at 4°C and used for the estimation of superoxide dismutase (SOD) and peroxidase (POX) activities. Protein concentration was determined by Lowry’s (1951) [86] method using BSA as a standard.

### Analysis of antioxidant enzymes activity

#### Guaiacol peroxidase enzyme assay

Peroxidase enzyme activity in the protein extract was measured by using the protocol proposed by Castillo *et al*. (1984) [87] with slight modification. Peroxidase activity was assayed as an increase in optical density due to the oxidation of guaiacol to tetra-guaiacol. Formation of tetra-guaiacol was measured at a time interval of 30 sec up to 1 min at 470 nm. One unit of peroxidase was defined as the amount of enzyme that oxidizes 1 μmole of guaiacol per min per gram fresh weight or per mg protein under standard assay condition.

Native PAGE was performed as the method described by Davis (1964) [88] by using 10% resolving gel and 5% stacking gel. Protein extract of 25 μg from all treatments was loaded into wells. Electrophoresis was conducted at 4°C for about 3 h. Later the gel was stained for peroxidase isoenzymes.

The gel was incubated in a solution containing 0.1 M Potassium phosphate buffer (pH 6.1), 20 mM guaiacol and 5.55 mM 30% (H_2_O_2_) for 10–20 min until the bands appeared. Then the gel was washed with 7.5% acetic acid and 1% glycerol to stop the reaction. The isoenzyme bands appeared brick red in colour.

#### Super Oxide Dismutase (SOD) assay

The enzyme SOD is a metalloprotein, which catalyzes the dismutation of superoxide radical to H_2_O_2_ and molecular oxygen. It is considered to be a key antioxidant in aerobic cells and constitutes the first line of defense against reactive oxygen species (ROS).

SOD activity was measured by the method described by Dhindsa *et al*., (1981) [89] with slight modifications. SOD activity was assayed by its ability to inhibit photochemical reduction of nitro blue tetrazolium chloride. The test tubes containing assay mixture were incubated under light for 15 min, illuminated and non-illuminated reactions without enzyme extract served as blanks. Absorbance of samples along with the blank was recorded at 560 nm wavelength. SOD activity is calculated as μg protein required for 50% inhibition of photochemical reduction of NBT.

Native PAGE was performed as the method described by Davis (1964) [88] by using 10% resolving gel and 5% stacking gel. Protein extract of 25 μg from all treatments was loaded into wells. Electrophoresis was conducted at 4°C for about 3 h. Later the gel was stained for super oxide dismutase isoenzymes.

The gel was incubated under a light in a staining solution containing 2.25 mM NBT (w/v), 3 mM EDTA, 0.1 M sodium phosphate buffer (pH 7.5), 15 μL TEMED and *5%* riboflavin (w/v) for 30 min until the bands appeared. The isoenzyme bands appeared colourless in a dark blue background and the isoenzyme pattern was photographed.

#### Analysis of secreted bioactive compounds by groundnut calli when exposed to SW-AgNPs

Liquid MS medium containing 1 mg/L 2, 4-D was used to obtain suspension cultures from groundnut calli. Media was prepared with or without SW-AgNPs. A packed cell volume of 200 μL of the calli was then inoculated into 50 mL of the liquid MS media and incubated in an orbital shaking incubator at 22°C and 80 rpm for 25 days.

After 25 days of incubation, the culture was transferred to 50 mL centrifuge tubes and centrifuged at 5000 rpm for 10 minutes and supernatant was collected and concentrated in a rotary vacuum evaporator. The concentrated content was mixed with membrane filtered methanol, and then the methanolic phase was collected for further analysis. The methanolic extract was passed again through the 0.2 micron membrane filter and the components were then analysed in a GC-MS model—QP2010 Shimadzu. A Column (SH-RXI-5SIL MS) of 30 m length, 0.25 mm inner diameter and column oven temperature of 50°C–280°C was used for the analysis. Samples were prepared with methanol and injected with a split mode in a 1: 100 ratio. Carrier gas was helium and blank solvent was methanol.

### Evaluation of anticancer effects of SW-AgNPs using cervical cancer cell lines

#### Cervical cell lines, reagents and culture conditions

Human cervical cancer cell lines-SiHa and CaSki cells were cultured in Dulbecco’s modified Eagle’s medium supplemented with 10% fetal bovine serum, and penicillin 100 units/mL and streptomycin 100 μg/mL. Normal cervical keratinocyte cell line HCK1T (obtained from NCCRI, Japan) were grown in a medium containing 3:1(v/v) F-12 Nutrient mixture: Dulbecco’s modified Eagle’s medium supplemented with 5% fetal bovine serum (FBS), hydrocortisone 0.4 μg/mL, insulin 5 μg/mL, cholera toxin 8.4 ng/mL, epidermal growth factor 10ng/mL, adenine 24 μg/mL, Y-27632 5–10 μmol/L. All cell lines were incubated in a 5% CO_2_ incubator at 37°C and cell lines were maintained by regular passage.

#### Exposure of cervical cancer cell lines to SW-AgNPs

Cervical cells (cancer cell lines—SiHa and CaSki, normal cervical cell—HCK1T) were used for evaluating the anti-cancerous activity of SW-AgNPs. Initial experiments were carried out with a broad range of concentrations starting from 0.1 ppm up to 200 ppm of SW-AgNP. Cells were incubated with the various concentrations of SW-AgNPs for 24 hours in all experiments. Controls including water control, media control, only plant extract, commercial AgNPs, autoclaved SW-AgNPs, citrate buffer (commercial AgNP is suspended in citrate buffer) were also used in the experiments to evaluate specificity of the effects.

#### Treatment details for cervical cell experiment

Single cell suspension of the three cervical cell lines were obtained by treatment with Trypsin EDTA on the adherent cells to release them from the bottom of the tissue culture flask and cells were collected by centrifugation. Cell pellet was resuspended in 1 ml of media and counted by trypan blue staining using a haemocytometer. All the three cell lines were seeded at a concentration of 5000 cells per well in a 96 well plate with their respective culture media and supplements. Plates were incubated in a humidified atmosphere containing 5% CO_2_at 37°C allowing the cells to attach and grow for 24 hours. Subsequently, medium was removed carefully from the wells and cells were exposed to different concentration SW-AgNPs in their respective culture media in triplicates. Controls such as water, plant extract, AgNO_3_, commercial AgNPs and autoclaved SW-AgNPs were included in the experiments.

#### Morphological analysis

Changes in the morphology of cervical cells incubated with various concentrations of SW-AgNPs were analysed my microscopy 24 hours post treatment. Bright field images (20x) of the cells were captured using Eclipse TE2000 Inverted Microscope (Nikon).

#### Analysis of cytotoxicity by MTT

(3-[4,5-dimethylthiazol-2-yl]-2,5 diphenyl tetrazolium bromide) **assay**: Cytotoxicity of SW-AgNPs was examined by following the protocol by Kumar *et al*., 2018 [90]. In brief, 100 μL of MTT solution (0.5mg MTT/mL of PBS) was added to each well of cells post the 24-hour treatment with the various concentrations of SW-AgNPs and incubated for 4 hours at 37°C. MTT solution was removed and 50 μL of DMSO solution was added to each well and mixed properly to dissolve the formazan crystals. The absorbance of each well was measured at 540 nm in a microplate reader. Percentage cell viability in each treatment was calculated by comparing with the media control and cytotoxicity effects of silver nano particles was expressed as IC_50_.

#### Analysis of cell viability by Neutral red uptake (NRU) assay

Cell viability and cytotoxic effect of SW-AgNPs was examined by following a protocol by Repetto *et al*., 2008 [91]. After 24 hours of treatment with the various concentrations of SW-AgNPs, medium was carefully removed and proceeded for Neutral red uptake assessment (Neutral Red medium- prepared by taking 1:100 dilution of Neutral Red stock solution in cell culture media. Stock solution- 40 mg neutral red/10mL of PBS. The media was prepared a day before use, centrifuged at 1800 rpm for 10 minutes to remove any precipitated crystals). 100 μL of centrifuged neutral red medium was added to each well. Culture plates were incubated at 37°C for 2 hours. After the incubation, neutral red medium was removed and cells are washed with PBS. 150 μl of Neutral Red distaining solution (Neutral Red distaining solution- 10 ml water, 10 ml ethanol 96% and 0.2 ml glacial acetic acid) was added per well and plates were kept on a shaker for 10 minutes. The absorbance of each well measured at 540 nm in a microplate reader and per cent cell viability of each treatment was calculated by comparing with the media control and cytotoxicity effects of SW-AgNPs was expressed as IC_50_.

#### Analysis of DNA damage in response to SW-AgNPs

Single cells of the three cervical cell lines were obtained by treatment with Trypsin EDTA on the adherent cells to release them from the bottom of the tissue culture flask and cells were collected by centrifugation. Cell pellet was resuspended in 1 mL of media and counted by trypan blue staining using a haemocytometer. All three cell lines were seeded at a concentration of 5×10^5^ cells in 60 mm culture dishes with their respective culture media and plates were incubated in a humidified atmosphere containing 5% CO_2_at 37°C allowing the cells to attach and grow for 24 hours. Subsequently, the media was removed carefully from the wells and cells were exposed to various concentration SW-AgNPs in their respective culture media along with the controls. Treatment media and cells washed with the PBS were collected to not lose any sample. Further, Trypsin EDTA was added to lift off the cells from the bottom of the plate and these cells were pooled with samples collected previously. The samples were centrifuged at 4000 rpm for 10 min and pellet was retained. In order to obtain the DNA, 50 μL of TES lysis buffer (20mM EDTA, 100mM Tris pH8 and 0.8% SDS) was added to the pellet and mixed by pipetting up and down. RNase A (2 μL) was added to the above solution and incubated at 37°C for 30 min. Proteinase K (5 μL) was added and incubated at 50°C for 90 min (based on protocol by Kasibhatla et al. 2006 [92]. DNA concentration was determined using nanodrop and normalized for 1 μg of DNA. Both 1 μg of DNA and an equal volume of DNA sample were loaded on to a 1.3% agarose gel dry wells separately and subjected to electrophoresis. DNA was visualized in gel documentation unit and imaged.

### Statistical analysis

Significance analysis between each group of data was carried out under the condition of P0.05. Origin 2022b software was used for the interpretation of UV-Vis, FTIR, and XRD peaks.

Microscopic cell counting for cell viability and Cells accumulating hydrogen peroxide were taken in 3 replications with 4 microscopic fields in each slide. For each of the treatments used, three replications were maintained. The statistical analysis of the callus culture data was carried out using Completely Randomised Design (CRD).

The MTT and neutral red uptake assays were carried out with triplicates for each of the treatments used and independent experiments were repeated at least three times independently.

Cell viability and IC_50_ value was calculated on Microsoft Excel and analysed further using GraphPad prism 8.0.1 by log transfoPrismon and non-linear regression curve fitting of the data.

## Supporting information

S1 Data (XLSX)
